# MTOPVIB interacts with AtPRD1 and plays important roles in formation of meiotic DNA double-strand breaks in *Arabidopsis*

**DOI:** 10.1038/s41598-017-10270-9

**Published:** 2017-08-30

**Authors:** Yu Tang, Zhongnan Yin, Yuejuan Zeng, Qinxin Zhang, Liqun Chen, Yan He, Pingli Lu, De Ye, Xueqin Zhang

**Affiliations:** 1 State Key Laboratory of Plant Physiology and Biochemistry, College of Biological Sciences, China Agricultural University, Beijing, 100193 China; 2 College of Agriculture and Biotechnology, China Agricultural University, Beijing, 100193 China; 3 School of Life Sciences, Fudan University, Shanghai, 200433 China

## Abstract

Meiotic recombination is initiated from the formation of DNA double-strand breaks (DSBs). In *Arabidopsis*, several proteins, such as AtPRD1, AtPRD2, AtPRD3, AtDFO and topoisomerase (Topo) VI-like complex, have been identified as playing important roles in DSB formation. Topo VI-like complex in *Arabidopsis* may consist of subunit A (Topo VIA: AtSPO11-1 and AtSPO11-2) and subunit B (Topo VIB: MTOPVIB). Little is known about their roles in *Arabidopsis* DSB formation. Here, we report on the characterization of the *MTOPVIB* gene using the *Arabidopsis* mutant alleles *mtopVIB*-*2* and *mtopVIB*-*3*, which were defective in DSB formation. *mtopVIB*-*3* exhibited abortion in embryo sac and pollen development, leading to a significant reduction in fertility. The *mtopVIB* mutations affected the homologous chromosome synapsis and recombination. MTOPVIB could interact with Topo VIA proteins AtSPO11-1 and AtSPO11-2. AtPRD1 interacted directly with Topo VI–like proteins. AtPRD1 also could interact with AtPRD3 and AtDFO. The results indicated that AtPRD1 may act as a bridge protein to interact with AtPRD3 and AtDFO, and interact directly with the Topo VI-like proteins MTOPVIB, AtSPO11-1 and AtSPO11-2 to take part in DSB formation in *Arabidopsis*.

## Introduction

In flowering plants, gametophyte formation relies on meiosis^1, 2^. Meiosis includes meiosis I and meiosis II. In meiosis I, the homologous chromosomes are separated into two daughter cells. In meiosis II, the sister chromosomes are then separated into newly formed daughter cells. As a result, each of the four newly formed daughter cells contains only a haploid set of chromosomes^3^. The prophase I of meiosis process can be divided into five stages: leptotene, zygotene, pachytene, diplotene and diakinesis. During prophase I, sister chromatid cohesion, homologous chromosome synapsis, recombination, crossover formation and chromosome segregation occur in a manner of continuous biological events, which ensure the normal formation of gametophytes^3, 4^.

Homologous recombination is initiated from the formation of DNA double-strand breaks (DSBs)^5^. In *Saccharomyces cerevisiae*, the formation of DSBs is catalyzed by Spo11^6^ with the assistance of at least other nine proteins^7–9^. The ten proteins are organized as four interacting subcomplexes, Spo11-Ski8, Rec102-Rec104, Rec114-Mei4-Mer2 and Mre11-Rad50-Xrs2^9^. Spo11 is homologous to topoisomerase VIA (TOP6A) in archaea^10^ and widely found in fungi, invertebrates, mammals and plants^8, 9^. Spo11 catalyzes the formation of DSBs and therefore induces meiotic DSB formation^8^. Ski8 has two physiological roles. One is that Ski8 is located in the cytoplasm and is involved in RNA metabolism in vegetative cells^11^. The other is that during meiosis, Ski8 is relocalized from the cytoplasm to meiotic chromosomes, which requires Spo11^12^. Ski8 can directly interact with Spo11 and is required for the interaction of Spo11 with Rec104^12^. Ski8 has a WD propeller motif and may function as a scaffold protein in DSB complex assembly during meiosis^9, 12^. After its formation, the Spo11-Ski8 subcomplex recruits Rec102-Rec104 subcomplex to localize Spo11 to chromatin sites and to bind to hotspots^13, 14^. Rec102-Rec104 localizes preferentially to chromatin loops and functions as a bridge to interact with Rec114-Mei4-Mer2 (RMM) to form a larger complex^14–16^. Mer2 is recruited to axial sites by Red1 and Hop1 (axis proteins), but independent of other DSB proteins^17, 18^. Mer2 is phosphorylated by cyclin-dependent kinase and is required for localization of Rec114 and Mei4 to axis sites^17–19^. RMM binds to chromosome axial sites, but DSBs occur at hotspots in chromatin loops^17, 20^. The mechanism of tethering these loop sites to the axis may explain this paradox^17, 20, 21^. Recent findings showed that Spp1 (a component of the COMPASS complex) can recognize and bind to H3K4me2/me3 marks on chromatin loops to axis sites through its direct interaction with Mer2^22, 23^. Finally, Mre11-Rad50-Xrs2 (MRX) complex is recruited during DSB formation and functions as a nuclease for DSB repair^24, 25^.

In mammals, SPO11, MEI1, MEI4 and REC114 have been identified as functioning in DSB formation. Mutation in *MEI1* causes sterility in mice and reduced γH2AX (a phosphorylated form of histone H2AX that is a marker for DSBs) signals. *MEI1* encodes an unknown function protein and is meiosis-specific in DSB formation^26, 27^. Recent findings showed that MEI1 is required for recruiting MEI4 to chromosome axes and that MEI4 directly interacts with REC114^28, 29^.

In *Arabidopsis*, six proteins, namely AtSPO11-1, AtSPO11-2, AtPRD1, AtPRD2, AtPRD3 and AtDFO, were found as being involved in DSB formation^30–34^. AtPRD1 is homologous to MEI1 of humans and mice^26, 27, 32^. It has a conserved N-terminal region that can interact with AtSPO11-1 and itself^32^. AtPRD2 is homologous to Mei4^33, 35^. AtPRD3 is a plant-specific protein for DSB formation and is the ortholog of the rice PAIR1^33, 36^. AtDFO is also a plant-specific protein with conserved function-unknown domains^34^. Studies have shown that AtSPO11-1 and AtSPO11-2 are involved in DSB formation^30, 31^, but how the *Arabidopsis* Topo VI-like complex interacts with other proteins involved in DSB formation remains unclear.

Recently, it was reported that TOPVIB-like proteins, which are homologous to archaeal Topo VIB proteins, are required for DSB formation in *Arabidopsis* (MTOPVIB)^37^, mice^38^ and rice^39, 40^. Here, we report our independent characterization of the *MTOPVIB* gene using the mutant alleles *mtopVIB*-*2* and *mtopVIB*-*3*. *mtopVIB*-*3* exhibited reduced fertility, polyads and abnormal tetrads. It had a weaker meiotic phenotype compared to *mtopVIB*-*2*. MTOPVIB could interact with AtSPO11-1 and AtSPO11-2. AtPRD1 could interact with the Topo VI-like complex proteins (MTOPVIB, AtSPO11-1 and AtSPO11-2). AtPRD1 may act as a bridge protein to recruit AtPRD3 and AtDFO to form a subcomplex, and may interact directly with Topo VI-like complex to promote meiotic DSB formation in *Arabidopsis*.

## Results

### Isolation of *mtopVIB*-3 mutant

**Figure 1:**
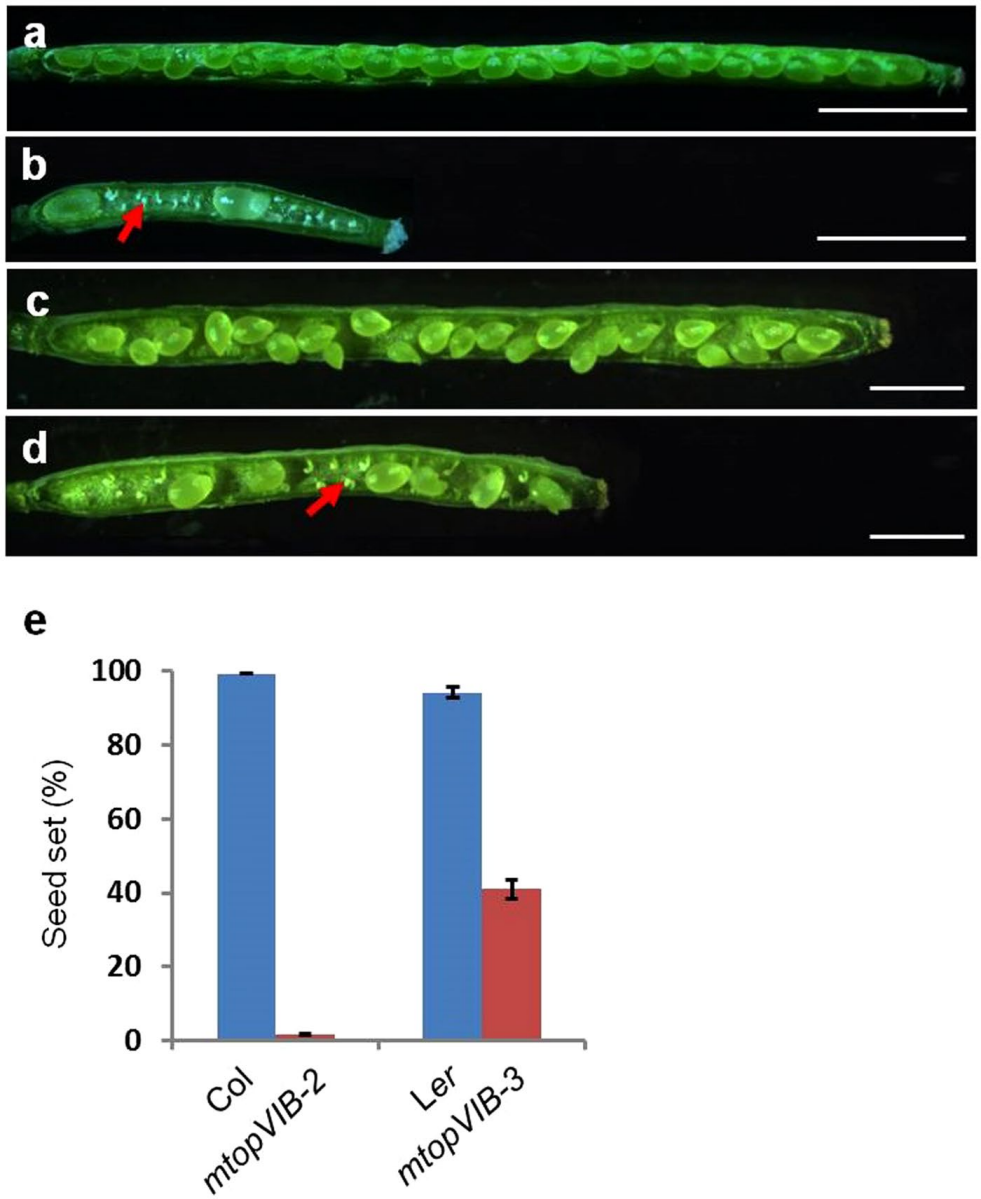
The *mtopVIB* mutants reduced fertility. (**a**) The wild-type (Col) silique with full seed set. (**b**) The *mtopVIB*-*2* silique with reduced seed set. (**c**) The wild-type (L*er*) silique with full seed set. (**d**) The *mtopVIB*-*3* silique with reduced seed set. (**e**) A comparison of seed set in wild-type, m*topVIB*-*2* and *mtopVIB*-*3* plants. The red arrows indicate the aborted ovules in the *mtopVIB* mutant siliques. Bars = 1 mm.

*mtopVIB*-*3* was identified as *mt187* in a screening of the enhancer- and gene-trap *Dissociation* (*Ds*) insertion lines in *Arabidopsis* ecotype Landsberg *erecta* (L*er*)^41^ by its sterility, which was not associated with the *Ds* insertion. Mapping-based cloning^42^ showed that the sterile phenotype was associated with a mutation in *At1g60460*. Meanwhile, a T-DNA-inserted allele bearing the sulfadiazine (SD) resistance reporter in the Col background was obtained from the *Arabidopsis* Biological Resource Center (ABRC, http://www.biosci.ohio-state.edu/pcmb/Facilites/abrc/abrchome.htm). When *At1g60460* was published as *MTOPVIB*, the *mt187* and Col alleles were renamed as *mtopVIB*-*3* and *mtopVIB*-*2* based on their being allelic to the mutant *mtopVIB*-*1* and identical to *mtopVIB*-*2* ^37^, respectively. *mtopVIB*-*2* and *mtopVIB*-*3* exhibited significantly reduced fertility and produced shorter siliques (Fig. 1a–d) with average one seed per silique (2% seed set; n = 6332) and average twelve seeds per silique (41% seed set; n = 2546) seed set, respectively (Fig. 1e). The progeny seedlings from the self-pollinated heterozygous *mtopVIB*-*2* mutant (*mtopVIB*-*2*/+) segregated at a ratio of 2.90 (1869) sulfadiazine-resistant (SD^R^) to 1 (645) sulfadiazine-sensitive (SD^S^). When *mtopVIB*-*2*/+ was used as female or male to cross with the wild-type plant, the segregation ratio of the progeny was 0.96 (652) SD^R^ to 1 (681) SD^S^ and 0.97 (1440) SD^R^ to 1 (1449) SD^S^, respectively (Supplementary Table S1). These results suggested *mtopVIB*-*2* was a sporophytic mutant.

### Mutation in *MTOPVIB* affects embryo sac development

**Figure 2:**
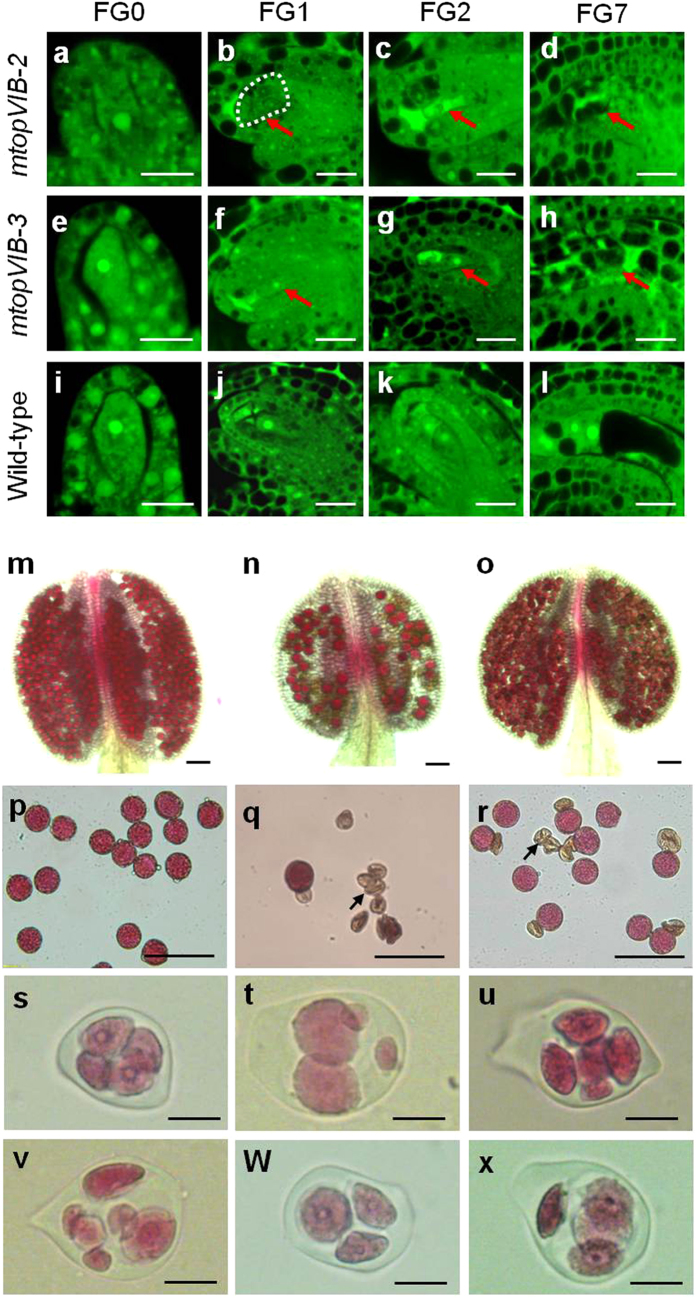
The *mtopVIB* mutants were defective in embryo sac and pollen development. (**a**–**d**) The ovules from an *mtopVIB*-*2* plant, showing the embryo sacs at stages FG0 (**a**), FG1 (**b**), FG2 (**c**) and FG7 (**d**), respectively. (**e**–**h**) The ovules from an *mtopVIB*-*3* plant, showing the embryo sacs at stages FG0 (**e**), FG1 (**f**), FG2 (**g**) and FG7 (**h**), respectively. (**i**–**l**) The ovules from wild-type plants, showing the embryo sacs at stages FG0 (**i**), FG1 (**j**), FG2 (**k**) and FG7 (**l**), respectively. (**m**) An Alexander-stained wild-type anther filled with viable pollen grains. (**n**–**o**) The Alexander-stained anthers from *mtopVIB*-*2* (**n**) and *mtopVIB*-*3* (**o**) plants, showing less viable pollen grains. (p-r) Pollen grains from wild-type (**p**), *mtopVIB*-*2* (**q**) and *mtopVIB*-*3* (**r**) plants. (**s**) A wild-type tetrad, (**t**–**v**) the polyads from *mtopVIB*-*2*: irregular tetrad (**t**), pentad (**u**) and hexad (**v**). (**w-x**) The polyads from *mtopVIB*-*3*: triad (**w**) and irregular tetrad (**x**). The red arrows indicate the abnormal nuclei, and dotted lines indicate the putative site of nucleus in *mtopVIB*-*2*. The black arrows indicate the abnormal pollen grains. Bars = 20 μm in (**a**–**r**), 50 μm in (**s**–**x**).

The *mtopVIB* ovules at different stages (Fig. 2a–h) were compared to those in the wild-type plant (Fig. 2i–l) using laser scanning confocal microscopy (LSCM)^43^. At the developmental stage FG0, the *mtopVIB*-*2* and *mtopVIB*-*3* ovules appeared normal as in the wild-type plant (Fig. 2a,e,i). At the FG1 stage, the unique nuclei of functional megaspores appeared smaller in *mtopVIB*-*3*, and no nucleus was observed in the *mtopVIB*-*2* embryo sacs (Fig. 2b,f), compared to those in the wild-type plant (Fig. 2j). At the FG2 stage, the two-nucleate embryo sacs in *mtopVIB*-*2* were not observed, instead, strong auto-fluorescent signals found in the mutant embryo sacs (Fig. 2c). In *mtopVIB*-*3*, single-nucleate embryo sacs were observed (Fig. 2g), compared to the wild-type embryo sacs, which had two nuclei after the first nuclear division of the functional megaspore (Fig. 2k). At the developmental stage FG7, the embryo sacs in *mtopVIB*-*2* and *mtopVIB*-*3* did not have the typical nuclear and cellular organization/structure, and they had only strong auto-fluorescent signals instead (Fig. 2d,h), compared to a typical four-celled mature embryo sac in wild-type plants (Fig. 2l), indicating that the embryo sacs in *mtopVIB* mutants had stopped developing and were degenerated. In particular, the *mtopVIB*-*2* and *mtopVIB*-*3* mutants had only 2.80% (n = 283) and 42.5% (n = 246) normal embryo sacs, respectively, compared to 99.3% (n = 133) in wild-type plants. The results indicated that the mutations in *MTOPVIB* showed abnormal embryo sac formation.

### Mutation in *MTOPVIB* affects pollen formation

SEM showed that the abnormal pollen grains from the mutants exhibited irregular shapes and sizes (Supplementary Fig. S1a–c), indicating abnormal pollen formation in *mtopVIB* mutants. Alexander staining was further used to examine wild-type (Fig. 2m) and *mtopVIB* mutants (Fig. 2n,o) pollen grains in the anthers. Only 0.5% (n = 4005) wild-type pollen grains were abnormal (Fig. 2P, Supplementary Fig. S1d), whereas 94.3% (n = 3082) and 37.7% (n = 4420) pollen grains were abnormal in *mtopVIB*-*2* and *mtopVIB*-*3* (Fig. 2q–r, Supplementary Fig. S1d), respectively. The defects were evident at the tetrad stage. Compared to the normal tetrads (n = 240) in the wild-type plant (Fig. 2s), the meiotic products were extremely irregular in both *mtopVIB* mutants, which had two to six spores or appeared as irregular tetrads (Fig. 2t–x). In *mtopVIB*-*2*, 95% of the abnormal polyads (n = 333) were irregular tetrads, pentads and hexads (Supplementary Fig. S2). In *mtopVIB*-*3*, 44% tetrads (n = 352) were abnormal (Supplementary Fig. S2). These results indicated that *mtopVIB*-*2* and *mtopVIB*-*3* mutants were defective in tetrad formation.

### *mtopVIB*-*2 and mtopVIB*-*3* mutants are defective in meiosis

To investigate whether the defect in tetrad formation was caused by abnormal meiosis in the *mtopVIB*-*2* and *mtopVIB*-*3* mutants, the chromosome behaviors in the mutant male meiocytes were examined by comparison with those of the wild-type plant. At the leptotene stage in the wild-type plant, the chromosomes appeared as single strands. The chromosomes then underwent synapsis from the zygotene to pachytene stages, which appeared as thick chromosome threads (Supplementary Fig. S3a–c). In the *mtopVIB*-*2* and *mtopVIB*-*3* male meiocytes, the chromosome behaviors displayed no obvious differences at the leptotene and zygotene stages (Supplementary Fig. S3e,f,i,j). At the pachytene stage, the chromosomes in the wild-type plant were shorter and thicker, whereas in the *mtopVIB*-*2* mutants, the chromosomes appeared thinner, and in the mutant *mtopVIB*-*3*, the chromosome structure was found with a weaker phenotype than that in *mtopVIB*-*2* (Supplementary Fig. S3c,g,k). Five normal pairs of highly condensed chromosome bivalents were observed in the male meiocytes of the wild-type plant at the diakinesis stage. At metaphase I, wild-type meiocytes had five well-aligned bivalents at the equatorial plate leading to equal chromosome segregation at the anaphase I stage (Supplementary Fig. S3d,m,n). In the *mtopVIB*-*2* and *mtopVIB*-*3* mutants, most chromosomes were appeared as univalents in the meiocytes at diakinesis. At metaphase I, compared to 5 bivalents (n = 89) in wild-type meiocytes, *mtopVIB*-*2* showed no bivalent, but 10 univalents (n = 90), and *mtopVIB*-*3* showed average number 3.2 of bivalents per meiocyte (n = 74), leading to improper chromosome segregation at anaphase I (Supplementary Fig. S3h,l,r,w,s,x). At metaphase II, the meiocytes in the wild-type plant had two sets of aligned chromosomes, and finally segregated to form four nuclei, each of which contained five chromosomes (Supplementary Fig. S3o,p). In the *mtopVIB*-*2* and *mtopVIB*-*3* mutant meiocytes at the same stage, misaligning of chromosomes was observed, and chromosome segregations at the anaphase II stage were then abnormal (Supplementary Fig. S3t,u,y,z). As a result, the *mtopVIB*-*2* and *mtopVIB*-*3* meiocytes generated four or more abnormal daughter cells and formed irregular tetrad and polyads after meiosis, instead of the normal tetrad (Supplementary Fig. S3q,v,a2).

In female meiosis, the mutant chromosomes at the leptotene stage were as normal as those in the wild-type plant (Supplementary Fig. S4a,e,i). At the pachytene stage, thick chromosome threads were not observed in *mtopVIB*-*2* and *mtopVIB*-*3* mutant ovules, as in the wild-type plant (Supplementary Fig. S4b,f,j). Moreover, at the metaphase I stage, the five bivalents were aligned on the metaphasic plate in the wild-plant (n = 77). In contrast, no bivalents in *mtopVIB*-*2* (n = 85) and average number 2.4 of bivalents per meiocyte in *mtopVIB*-*3* female meiocytes (n = 83) were observed (Supplementary Fig. S4c,g,k). At the telophase I stage, chromosomes in the female mutant meiocytes were also segregated unequally like those in the male meiocytes (Supplementary Fig. S4d,h,l). Taken together, these results indicated that the mutations in *MTOPVIB* drastically affected both male and female meiotic division.

Co-immunolocalization of ASY1 and ZYP1 was determined to investigate whether synapsis was defective in *mtopVIB*-*2* and *mtopVIB*-*3*. ASY1 is associated with the chromosome axes^44^, while ZYP1 is a major component of the central element of the synaptonemal complex (SC)^45^. The ASY1 signals showed no obvious differences between the wild-type and mutant meiocytes at the pachytene stage (Supplementary Fig. S5a,b,e,f,i,j). In contrast, no ZYP1 signals in *mtopVIB*-*2* and weak ZYP1 signals in *mtopVIB*-*3* were observed compared to those in wild-type meiocytes (Supplementary Fig. S5c,d,g,h,k,l). The results showed that synapsis did not occur in *mtopVIB*-*2* and was clearly reduced in *mtopVIB*-*3* meiocytes.

### *mtopVIB*-*3* is caused by a point mutation in *MTOPVIB*

Genetic analysis showed that the F1 progeny from the self-pollinated heterozygous *mtopVIB*-*3* mutant plants segregated in an approximate ratio of 3 normal:1 abnormal (172:68), implying that *mtopVIB*-*3* might be a single, recessive nuclear mutation. To identify the mutation point, the *mtopVIB*-*3* mutant in L*er* background was used as a female to cross with the wild-type Col plant. F2 plants with the sterile phenotype and without *Ds* insertion were selected and used for map-based cloning. *mtopVIB*-*3* was initially mapped to the bottom arm of chromosome 1 and was then precisely mapped in between the BAC clones T13D8 and F8A5 (Supplementary Fig. S6a). Sequencing of all the genes in the region between BAC T13D8 and F8A5 revealed three notable mutations, of which two, A693G and A800G (counting from the first putative start codon ATG), were the same as those in the wild-type Col genome in *AT1G60450*. The other, G1890A (counting from the first putative start codon ATG), was located at the last nucleotide of the eighth exon of *AT1G60460* (*MTOPVIB*) in *mtopVIB*-*3* (Supplementary Fig. S6b), which was not found in the wild-type genome. Therefore, we speculated that G1890A in *AT1G60460* might be related to the phenotype of *mtopVIB*-*3*.

RT-PCR assays using the RNAs from *mtopVIB*-*3* and wild-type inflorescences showed that only the expression of *AT1G60460* was detected as being changed among the ten genes in the region between BAC T13D8 and F8A5 of *mtopVIB*-*3*, compared to those in the wild-type plant (Supplementary Fig. S6c). The real-time PCR assay showed that the expression level of *mtopVIB*-*3* was reduced to 0.51-fold of the wild-type expression level (Supplementary Fig. S6d). Monoclonal sequencing of the RT-PCR products indicated that the *MTOPVIB* RNAs were changed in *mtopVIB*-*3*. Seven wild type *MTOPVIB* transcripts showed two types: five normal transcripts and two transcription intermediates containing the 136-bp intron behind the eighth exon (Table 1; Supplementary Fig. S6e). The sequences of the thirteen RT-PCR products from *mtopVIB*-*3* could be catalogued into four different types (Table 1). First, five transcripts contained the 136-bp intron behind the eighth exon (the point mutation site), which could be the transcription intermediates like those detected in wild-type, might encode 493 aa (221^R-K^) (Supplementary Fig. S6f). Second, four transcripts lacked the 43-bp sequence upstream of the mutation site. Third, one transcript had an extra 32 bp behind the mutation point. Fourth, two transcripts had an extra 11 bp behind the mutation point (Supplementary Fig. S6e). In the last three cases, new GU-AG intron splicing sites were identified as being different from the primary splicing site of the eighth exon in the wild-type plant, and might cause early termination of translation. These results implied that the point mutation dramatically affected the splicing of the *MTOPVIB* RNAs in *mtopVIB*-*3* mutant. The change in splicing sites that led to truncation of the MTOPVIB protein and might cause a leaky phenotype in *mtopVIB*-*3* (Supplementary Fig. S7).

**Table 1:** The point mutation in *mtopVIB*-*3* affected the splicing of the *MTOPVIB* RNAs.

Plants	Samples	Sequencing of transcripts
L*er*-*0*	5 (7)	correct transcript
L*er*-*1*	2 (7)	intermediate transcripts with an extra 136-bp intron
*mtopVIB*-*3*-*1*	5 (13)	intermediate transcripts with an extra defective 136-bp intron
*mtopVIB*-*3*-*2*	4 (13)	−43 bp
*mtopVIB*-*3*-*3*	1 (13)	+32 bp
*mtopVIB*-*3*-*4*	2 (13)	+11 bp

*mtopVIB*-*3*-*1* containing 136-bp intron might translate 493 aa with 221^R-K^. *mtopVIB*-*3*-*2* lost 43 bp up to the point mutation. *mtopVIB*-*3*-*3* and -*4* contained 32-bp and 11-bp intron after the point mutation, respectively. The last three cases might cause early termination of translation.

In *mtopVIB*-*2*, the T-DNA was inserted in the fifth intron of *MTOPVIB*. No normal *MTOPVIB* transcript was detected in the homozygous *mtopVIB*-*2* plants (Supplementary Fig. S6g–i). The T-DNA insertion also disfunctioned the MTOPVIB protein (Supplementary Fig. S7).

To confirm that the *mtopVIB*-*3* phenotype was caused by the point mutation (G1890A) in *MTOPVIB*, a 5.959-kb full-length genomic DNA fragment of *MTOPVIB*, including 2.382-kb promoter region, 3.577-kb coding region and 289-bp 3′-terminal region, was cloned into the pCAMBIA1300 vector (CAMBIA, Canberra, Australia) and then introduced into the *mtopVIB*-*3* plants by *Agrobacterium*-mediated infiltration. In total, 14 independent transgenic plants were obtained, 12 of which had full seed set restored like that in the wild-type plant (Supplementary Fig. S8a). All the siliques from these transgenic *mtopVIB*-*3* plants were morphologically normal and had full seed set (Supplementary Fig. S8b). The siliques from complemented *mtopVIB*-*3* plants had a similar length as that of the wild-type plant (Supplementary Fig. S8b,c). In particular, the seed set rate in the complemented *mtopVIB*-*3* plants was restored to 97.7% (n = 663), close to 97.9% (n = 512) of the wild-type plant, compared to 41.7% (n = 510) in the non-transgenic *mtopVIB*-*3* plants (Supplementary Fig. S8d). This result showed that the cloned genomic fragment could encode the full functions of the MTOPVIB protein. Thus, the meiotic defect in the *mtopVIB*-*3* plants was caused by the mutation in *MTOPVIB*.

Real-time PCR assays showed that *MTOPVIB* was expressed ubiquitously in different tissues, including roots, stems, leaves, seedlings, inflorescences, siliques and pollen grains, specially as a higher level in inflorescences (Supplementary Fig. S9a). MTOPVIB protein localized on chromosomes as described by Vrielynck *et al*.^37^. By fusion of the MTOPVIB protein with the reporter GFP under the control of the cauliflower mosaic virus (CaMV) 35 S promoter, strong GFP-MTOPVIB signals were detected in the nuclei and weak ones in the cytoplasm (Supplementary Fig. S9e–g). The GFP signals in the control were distributed in the whole cell (Supplementary Fig. S9b–d). This result indicated that overexpression of MTOPVIB can lead to distribute GFP signals in nuclear and cytoplasm.

### The *mtopVIB* mutants are defective in meiotic DSB formation

The F1 plants from crosses of *mtopVIB*-*3* (L*er*) with *mtopVIB*-*2*/+ (Col) were used to analyze the chromosome recombination frequency. Two markers (F3P11 and T16B24) on chromosome II and two markers (T26D22 and K6M13) on chromosome V were used to measure the recombination frequencies between each of the two pairs. The recombination rate was 10.9% for chromosome II and 14% for chromosome V in *mtopVIB*-*2*/*mtopVIB*-*3*, compared to 21.4% and 30.5% in *mtopVIB*-*3*/+ plant (control) (Table 2), respectively, indicating that *mtopVIB*-*2* and *mtopVIB*-*3* had a significant reduction in recombination.

**Table 2:** Recombination frequencies in *mtopVIB*-*2*/*mtopVIB*-*3*.

Genotype	Chromosome II Recombination Frequency F3P11/T16B24	Chromosome V Recombination Frequency T26D22/K6M13
*mtopVIB*-*3*/+	21.4%	30.5%
*mtopVIB*-*2*/*mtopVIB*-*3*	10.9%	14.0%

Quantitative analysis of the recombination frequencies in both *mtopVIB*-*3/*+ (control) and *mtopVIB*-*2/mtopVIB*-*3* (experimental), using four sets of InDel markers on chromosome II and chromosome V, respectively.

**Figure 3:**
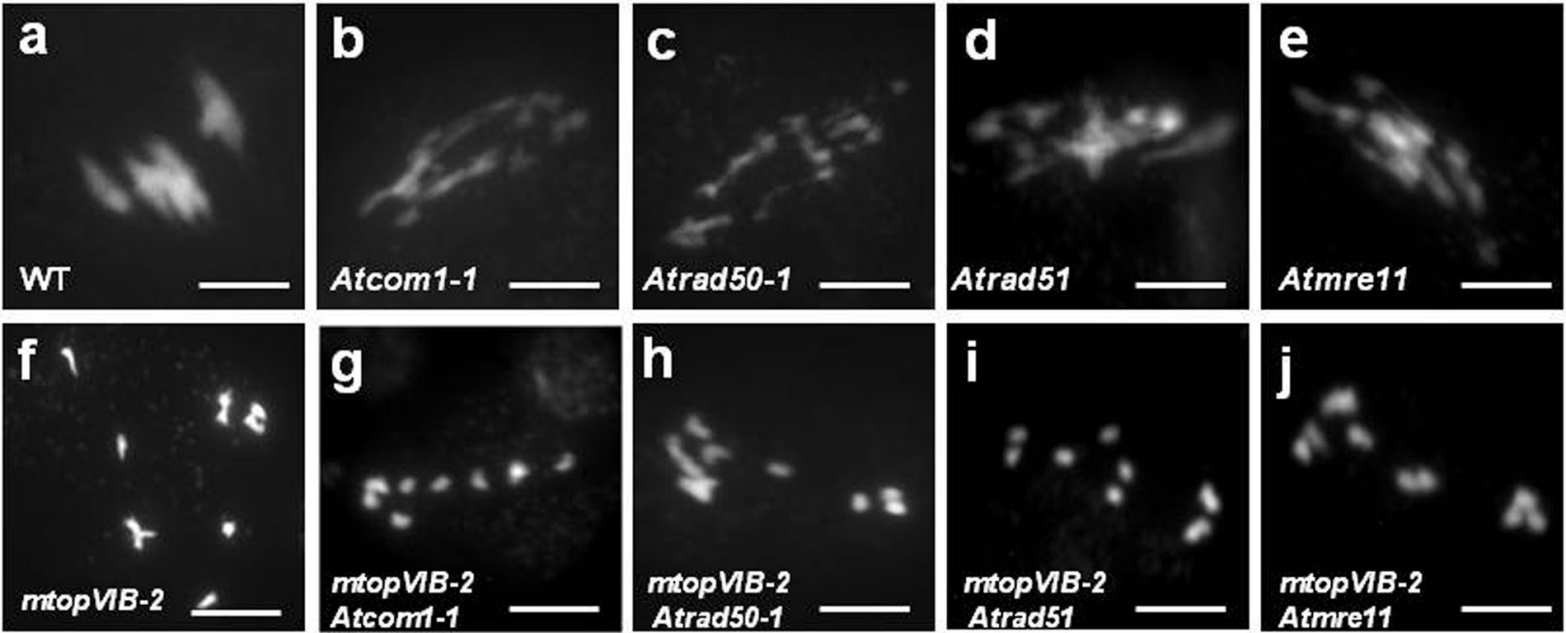
The *mtopVIB* mutants were defective in DSB formation. (**a**) A wild-type meiocyte with five bivalents at metaphase I. (b–e) The meiocytes at metaphase I from *Atcom1*-*1* (**b**), *Atrad50*-*1* (**c**), *Atrad51* (**d**) and *Atmre11* (**e**) that exhibited chromosome fragmentations. (**f**–**j**) The meiocytes at metaphase I from *mtopVIB*-*2* (**f**), *mtopVIB*-*2 Atcom1*-*1* (**g**), *mtopVIB*-*2 Atrad50*-*1* (**h**), *mtopVIB*-*2 Atrad51* (**i**) and *mtopVIB*-*2 Atmre11* (**j**) had ten univalents, in which no chromosome fragmentation was observed. Bars = 10 μm.

To investigate whether reduction in recombination was related to DSB formation or DSB repair, the strong allele *mtopVIB*-*2* was crossed with the four DSB repair-defective mutants *Atcom1*-*1*, *Atrad50*-*1*, *Atrad51*-*1* and *Atmre11*-*4* ^46–49^. These mutants do not affect the formation of DSBs, but are defective in repair of DSBs, leading to fragmentation of chromosomes. The double mutants of *mtopVIB*-*2 Atcom1*-*1*, *mtopVIB*-*2 Atrad50*-*1*, *mtopVIB*-*2 Atrad51*-*1* and *mtopVIB*-*2 Atmre11*-*4* had only ten univalents and no bivalents at metaphase I, like that in *mtopVIB*-*2* (Fig. 3a–j). Furthermore, no fragmentation of chromosomes was observed in these double mutants. The result indicated that the *mtopVIB* mutants were related to DSB formation.

### MTOPVIB interacts with AtPRD1

To further investigate whether MTOPVIB is associated with DSB formation in *Arabidopsis*, assays for the interactions of MTOPVIB with the known proteins that are involved in DSB formation were performed. Yeast two-hybrid (Y2H) and bimolecular fluorescence complementation (BiFC) assays were performed. Western blot was firstly to detect that these proteins were expressed in yeast cells (Supplementary Fig. S10a,b). Then using Y2H and BiFC to carry out protein interactions and found that MTOPVIB could interact with AtSPO11-1 and AtSPO11-2 (Supplementary Tables S2, S4; Fig. S11a–c). These results suggested that MTOPVIB (Topo VIB) could associate with AtSPO11-1 and AtSPO11-2 (Topo VIA) to form Topo VI-like complex.

**Figure 4:**
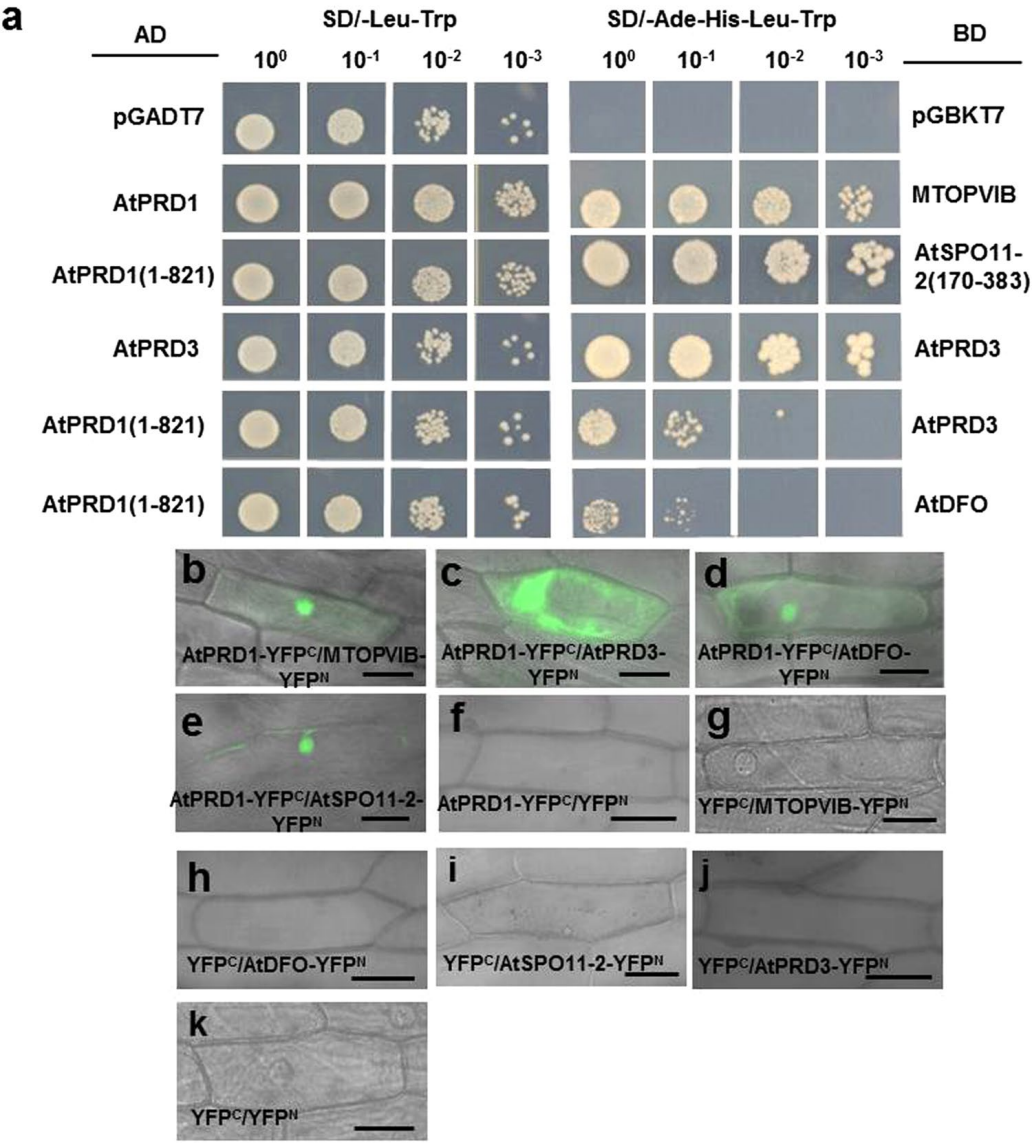
AtPRD1 interacted with MTOPVIB, AtSPO11-2 and AtPRD3, AtDFO. (**a**) The yeast two-hybrid assay showed that: (1) AtPRD1 interacted with MTOPVIB; (2) AtPRD1 (1-821 aa) interacted with AtSPO11-2 (170-383 aa); (3) AtPRD3 interacted with itself; (4) AtPRD1 (1-821 aa) interacted with AtPRD3 and AtDFO. The yeast cells were serially diluted in the cultures to evaluate the interaction. (**b**–**e**) The BiFC assay showed interactions of AtPRD1 with MTOPVIB (**b**), AtPRD3 (**c**), AtDFO (**d**) and AtSPO11-2 (**e**) in onion epidermal cells. (**f**–**k**) The negative controls for the (**b**–**e**), respectively. Bars = 50 μm.

Furthermore, the Y2H assay also showed that the truncated AtPRD1 (1-821 aa) which contained only the N-terminal region of AtPRD1 could interact with AtSPO11-1 and the full length AtPRD1 (1–1330 aa) (Supplementary Fig. S11a). The BiFC assay in onion epidermal cells also yielded similar results, indicating that AtPRD1 could interact with AtSPO11-1 (Supplementary Fig. S11d). AtPRD1 also could interact with MTOPVIB and AtSPO11-2 and AtPRD3 and AtDFO (Fig. 4a–e, Supplementary Table S2) in the two assay systems. Taken together, the results indicated that AtPRD1 may interact with MTOPVIB, AtSPO11-1, AtSPO11-2, AtPRD3 and AtDFO in DSB formation.

*AtPRD1* encodes a function-unknown protein with 1330 aa in *Arabidopsis*, and contains two potential coiled-coil structures in the C-terminal region (800–850 aa and 1280–1330 aa), which may be related to protein-protein interaction (https://npsa-prabi.ibcp.fr/cgi-bin/primanal_lupas.pl). To examine the roles of different parts of AtPRD1 in its interaction with MTOPVIB and other related proteins, the AtPRD1 protein was divided into five fragments: AtPRD1-A (1–388 aa), AtPRD1-B (389–812 aa), AtPRD1-C (813–962 aa), AtPRD1-D (963–1109 aa) and AtPRD1-E (1110–1330 aa) (Supplementary Fig. S12a). Meanwhile, the MTOPVIB protein was also truncated into two fragments: Bergerat domain (1–261 aa) and transducer domain (228–493 aa) (Supplementary Fig. S12b). In the Y2H and BiFC assays, both AtPRD1-A (1–388 aa) could interact with MTOPVIB, AtSPO11-1 and AtSPO11-2, AtPRD3 and AtDFO (Supplementary Tables S3, S4; Fig. S13a–e). Furthermore, AtPRD1-D (963–1109 aa) showed weak interactions with MTOPVIB, AtPRD3 and AtDFO (Supplementary Table S3; Fig. S11f–h), but AtPRD1-B, AtPRD1-C and AtPRD1-E could not interact with any of the proteins tested. These results implied that AtPRD1-A might be an important region for its interactions with MTOPVIB, AtSPO11-1, AtSPO11-2, AtPRD3 and AtDFO.

**Figure 5:**
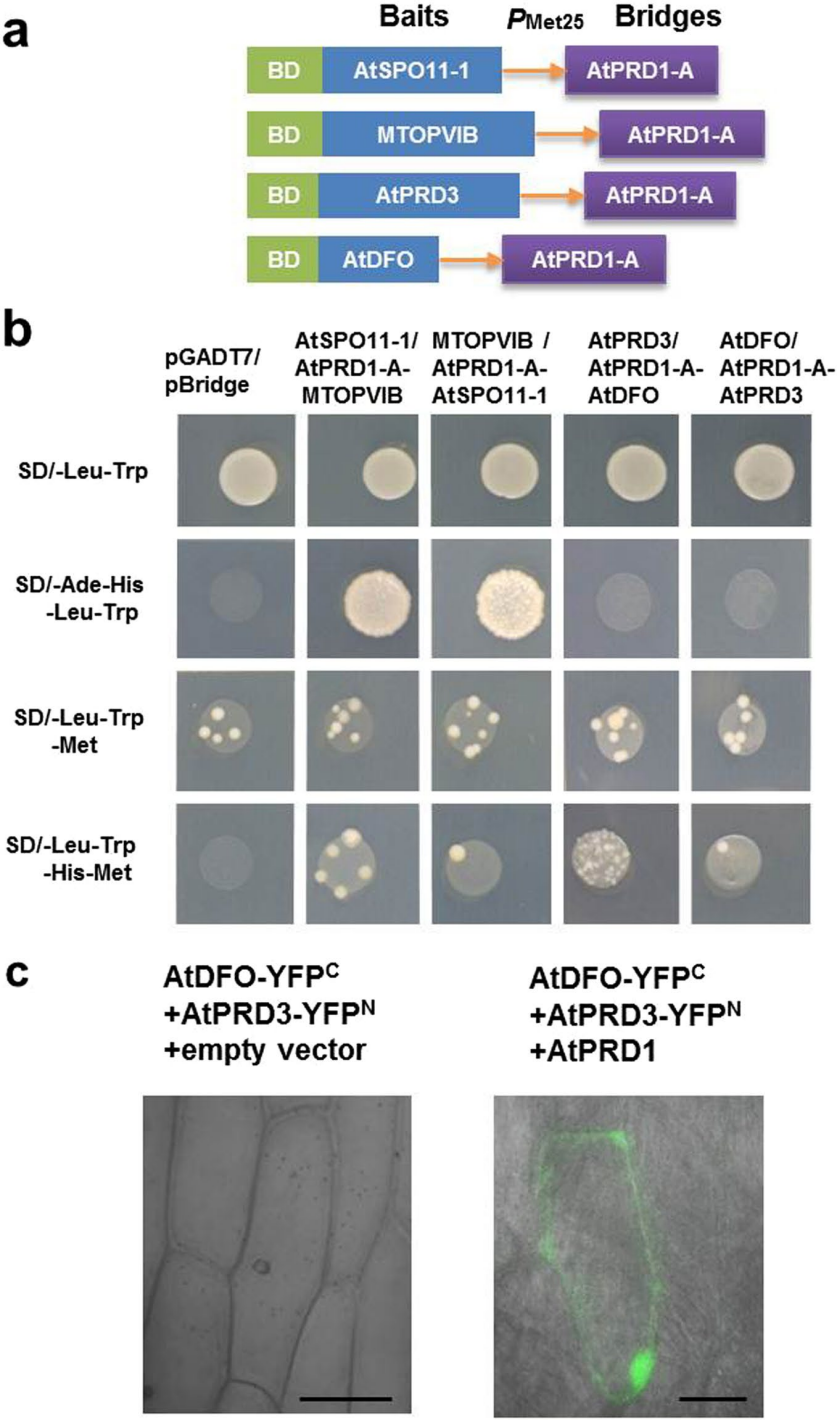
AtPRD1 might act bridge interaction with AtPRD3 and AtDFO. (**a**) The constructs expressing both the bait and the bridge proteins for Y3H assays. (**b**) The results of the Y3H assay. The yeast cells harboring pA-AtSPO11-1 and pB-AtPRD1 (1-388 aa)-MTOPVIB or pA-MTOPVIB and pB-AtPRD1 (1-388 aa)-AtSPO11-1 could grow well in SD/-Ade-His-Leu-Trp or SD/-Leu-Trp-His-Met media, indicating that AtPRD1 did not influence the interaction of MTOPVIB with AtSPO11-1. The yeast cells harboring pA-AtPRD3 and pB-AtPRD1 (1-388 aa)-AtDFO or pA-AtDFO and pB-AtPRD1 (1-388 aa)-AtPRD3 could grow in SD/-Leu-Trp-His-Met medium but not in SD/-Ade-His-Leu-Trp medium, indicating that AtPRD1 might function as a bridge between MTOPVIB, AtPRD3 and AtDFO. (**c**) BiFC assay indicated AtPRD1 might be a bridge protein to mediate the interaction of AtPRD3 and AtDFO. Bars = 50 μm.

The yeast three-hybrid (Y3H) assay was performed to examine the relationship of AtPRD1 with AtSPO11-1 and MTOPVIB using the combination of a prey construct expressing MTOPVIB and a bait construct expressing AtSPO11-1 and AtPRD1-A bridge protein (Fig. 5a). Alternatively, the assay was also performed using the combination of a prey construct expressing AtSPO11-1 and a bait construct expressing MTOPVIB and AtPRD1-A (1–388 aa) bridge protein (Fig. 5a). Under the conditions that AtPRD1-A (1-388 aa) was not expressed in the transgenic yeast cells grown in SD/-Leu-Trp-His-Ade medium, MTOPVIB could interact with AtSPO11-1. Under the conditions that induced the expression of AtPRD1-A (1-388 aa) in the transgenic yeast cells grown in SD/-Leu-Trp-His-Met medium, MTOPVIB also could interact with AtSPO11-1. The results suggested that AtPRD1-A (1-388 aa) expression did not affect the interaction of MTOPVIB with AtSPO11-1 in yeast (Fig. 5b).

The relationship of AtPRD1 with AtDFO and AtPRD3 was then examined using the combination of a prey construct expressing AtDFO and a bait construct expressing AtPRD3 and AtPRD1-A (1–388 aa) bridge protein, or the alternative combination of a prey construct expressing AtPRD3 and a bait construct expressing AtDFO and AtPRD1-A (1–388 aa) bridge protein (Fig. 5a). AtPRD3 and AtDFO could not interact with each other when the truncated AtPRD1-A was not expressed in the transgenic yeast cells grown in SD/-Leu-Trp-His-Ade medium, but they did when the truncated AtPRD1-A was expressed in the transgenic yeast cells grown in SD/-Leu-Trp-His-Met medium (Fig. 5b). Furthermore, the BiFC assay showed that AtPRD1 could mediate the interaction of AtPRD3 and AtDFO in onion epidermal cells (Fig. 5c). These results suggested that AtPRD1 did not influence the formation of Topo VI-like complex, but might act bridge interaction with AtPRD3 and AtDFO.

## Discussion

DSB formation is an important step of meiotic chromosome recombination that is widespread among plant sexual reproduction. Several proteins have been identified as being involved in DSB formation in *Arabidopsis*, including MTOPVIB, which was reported recently by Vrielynck *et al*.^37^. However, the roles of these proteins in DSB formation remain unclear. Furthermore, little is known about how these proteins work to promote DSB formation in plants including *Arabidopsis*. In this study, we independently investigated the roles of MTOPVIB in DSB formation by characterization of *mtopVIB* mutants and the MTOPVIB protein. *mtopVIB*-*3* was caused by point mutation in the last base pair (G1890) of the eighth exon in *MTOPVIB*. The mutation of G1890A caused alternated splicing of the related introns and disrupted the function of the gene, leading to significant reduction in fertility of the plants. However, some MTOPVIB proteins (493 aa (221)^R-K^) might exist and function normally in *mtopVIB*-*3*, which lead to *mtopVIB*-*3* showed leaky phenotype with some bivalents. The *mtopVIB*-*2* was a knockout allele which disrupted formation of bivalent. Genetic analyses showed that functional loss of *MTOPVIB* severely affected male and female gametophyte formation, leading to drastic reduction of seed set. The cytological characterization of the *mtopVIB* mutants suggested that MTOPVIB plays important roles in meiosis. These results are consistent with the data recently reported by Vrielynck *et al*.^37^.

This study demonstrated that MTOPVIB is involved in DSB formation instead of DSB repair. First, the *mtopVIB* mutants were found to be defective at the pachytene stage, implying that MTOPVIB is related to homologous chromosome synapsis and recombination. Furthermore, the assays using the synapsis marker proteins ASY1 (as lateral elements) and ZYP1 (as transverse elements)^44, 45^ showed that the *mtopVIB* mutant meiocytes were defective in synapsis, which is also similar to the characteristic of the mutants involved in DSB formation. In addition, the chromosome recombination frequencies were dramatically reduced in the *mtopVIB* mutants (Table 2), also suggesting that the *MTOPVIB* gene is related to DSB formation.

Recently, MTOPVIB was demonstrated as an essential component of the Topo VI-like complex that is involved in DSB formation^37^. In this study, our results further confirmed that MTOPVIB is involved in DSB formation, instead of DSB repair, as demonstrated by the double-mutant analysis of *mtopVIB*-*2* with the DSB repair-defective mutants *Atcom1*-*1*, *Atrad50*-*1*, *Atrad51*-*1* and *Atmre11*-*4* ^46–49^. Double mutants *mtopVIB*-*2 Atcom1*-*1*, *mtopVIB*-*2 Atrad50*-*1*, *mtopVIB*-*2 Atrad51*-*1* and *mtopVIB*-*2 Atmre11*-*4* all showed only ten univalents but no bivalents at the metaphase I stage, suggesting that MTOPVIB was not involved in DSB repair.

Our results also showed that *Arabidopsis* MTOPVIB could interact with AtSPO11-1 and AtSPO11-2, suggesting that they can interact with each other to form a Topo VI-like complex and catalyze DSB formation. A previous study showed that AtPRD1 is involved in DSB formation and that the N-terminal region of AtPRD1 could interact with itself and AtSPO11-1^32^. Our results showed that AtPRD1 also could interact with MTOPVIB and AtSPO11-2.

Furthermore, as demonstrated by Y2H and BiFC assays, AtPRD1 also interacted with AtPRD3 and AtDFO. AtPRD1 is homologous to human MEI1 in amino acid sequence, with a conserved domain in its N-terminal region^27, 32^. The structural analysis showed that AtPRD1 (46–576 aa) might contain putative armadillo repeats, which exhibited a strong similarity to β-catenin in human or importins in yeast (https://www.swissmodel.expasy.org/), and might mediate recognition and interaction with its partners. Furthermore, the truncation assays indicated that AtPRD1-A (1–388 aa) had a stronger affinity in the interaction with Topo VI-like complex, AtPRD3 and AtDFO, respectively. The AtPRD1 also contains coiled-coil domains that may be involved in protein-protein interactions. However, our truncation assays showed that coiled-coil-containing truncated fragments AtPRD1-C and AtPRD1-E could not interact with other proteins tested, suggesting that the coiled-coil domains in AtPRD1 may not be required for its interaction with other component proteins.

Y3H assay showed that AtPRD1 did not have any impact on the interaction of MTOPVIB and AtSPO11-1, suggesting that it did not influence the formation of Topo VI-like complex. Y3H and BiFC assays showed that AtPRD1 might be a bridge protein to interact with AtDFO and AtPRD3. Therefore, AtPRD1 may recruit AtPRD3 and AtDFO to form a subcomplex, and interact with Topo VI-like complex to promote DSB formation. In mice, MEI1 (the homologue of AtPRD1) could recruit MEI4 (the homologue of AtPRD2) to localize to axial sites^29^. However, in *Arabidopsis*, AtPRD1 did not interact with AtPRD2, but could directly interact with AtPRD3 and AtDFO and the Topo VI-like proteins. AtPRD1 may take part in recruiting these proteins and may affect the localization of these proteins. Nevertheless, more studies are required to verify the hypothesis.

## Methods

### Plant materials and growth conditions

All *Arabidopsis thaliana* materials used in this study are of Landsberg *erecta* (L*er*), Columbia (Col-0) and Wassilewskija (Ws) backgrounds. The *mtopVIB*-*3* mutant was isolated as described previously^41, 50^, and GK-314G09-015819 (*mtopVIB*-*2*) was obtained from the *Arabidopsis* Biological Resource Center (ABRC). The *mtopVIB*-*3* is backcrossed with wide-type plant (L*er*) three times to generate the purified *mtopVIB*-*3* without *Ds* insertion for further application. The other T-DNA insertion mutants used in this work, namely *Atrad51* (SAIL_873_C08), *Atrad50*-*1* (WiscDsLox402F12), *Atmre11* (SALK_067823) and *Atcom1*-*1* (SALK_061706C), were obtained from ABRC. All the *Arabidopsis* seeds were surface-sterilized and plated on Murashige and Skoog (MS) agar plates in cool (4–7 °C) conditions for 2–4 days. The plates were transferred to the growth room with 16 h light/8 h dark cycles at 22 °C for seed germination and further growth. The resulting 7-day-old seedlings were transplanted to soil for further growth under the same conditions.

### Phenotype analysis

The *Arabidopsis* wild-type and mutant plants were photographed with a Canon digital camera (Canon, Tokyo, Japan). Laser scanning confocal microscopy of ovules were performed as described previously^1^. The pollen grains and dissected tetrads were collected from wild-type and mutant plants, then stained with 4′,6-diamidino-2-phenylindole (DAPI) and Alexander solution as described previously^1^. Morphology of the pollen grains was observed using scanning electron microscopy (SEM, HITACHI, TM3000). Preparation of *Arabidopsis* meiotic chromosome spreads was performed as described previously^51^. The samples were stained with DAPI solution (0.1 M sodium phosphate, pH 7.0, 1 mM EDTA, 0.1% Triton X-100 and 0.25 mg/mL DAPI). The images of the chromosome spreads were captured using OLYMPUS CCDDP26 microscope (http://olympus-imaging.cn) and Leica DM2500 microscope (http://www.leica.com).

### Immunolocalization

Immunofluorescence was performed as previously described with minor modifications^52^. The primary antibodies were diluted at 1:200 (anti-ASY1) or 1:100 (anti-ZYP1) in blocking buffer (1 × PBS containing 0.1% Tween 20, 5% BSA and 1 mM EDTA) and added to the slides, followed by overnight incubation at 4 °C in a moisture chamber. The slides were washed with washing buffer (1 × PBS containing 0.1% Tween-20) three times (15 min each time). The secondary antibodies diluted at 1:400 in blocking buffer were added to slides, followed by incubation in a moisture chamber at 37 °C for 60 min in the dark. The slides were washed three times (15 min each) with washing buffer, and then stained with DAPI and observed using an OLYMPUS CCD DP26 fluorescence microscope.

### Mapping cloning and complementation experiment

The purified *mtopVIB*-*3* mutant plants were crossed with wild-type (Col) plants to generate hybrid plants. The individual F2 plants with sterile phenotype were selected from the 1500 mutant F2 plants and used for mapping cloning. Simple sequence length polymorphism (SSLP) markers (Supplementary Table S5) were used to map the mutation in *MTOPVIB*. The mutation was primarily located between the BAC clones F2J6 and T6C23 on chromosome 1. The further mapping using the markers of T30E16, T13D8, F8A5, F23C21, T7P1, F11P17 and F8K4 located the mutation position of *mtopVIB*-*3* in the region between the BAC clones T13D8 and F8A5. Finally, the mutation region covering 10 candidate genes was sequenced and then compared to the corresponding wild-type sequences from the GenBank database.

To perform *mtopVIB*-*3* complementation, the full-length *MTOPVIB* genomic DNA fragment (5.959 kb), including 2.382-kb promoter, 3.577-kb open reading frame and 289-bp 3′-terminal non-translation region, was amplified by PCR using high-fidelity DNA polymerase (Fast Pfu PCR kit, Product No. AP231, TaKaRa, Dalian, http://www.takara.com.cn, China) and the gene-specific primers (Forward: 5′-TGCGCCAAAGGAAAATGAAGA-3′; Reverse: 5′-CTTGCAGGGAAGTCACAAGA-3′). The resulting full-length DNA fragment was cloned into vector pMD19-T (Product No. D102A, TaKaRa, Dalian, China http://www.takara.com.cn). After verified by sequencing, the DNA fragment was excised by restriction enzymes KpnI and SalI and then subcloned into the binary vector pCAMBIA-1300 (CAMBIA, Canberra, Australia). The resulting construct was then transformed into *mtopVIB*-*3* mutant by the *Agrobacterium*-mediated infiltration method^53^. The transformants were screened on MS medium containing 25 mg/L hygromycin (Roche, Shanghai, China, http://www.roche.com.cn) and used for evaluation of the complementation by phenotypic and genetic characterization.

### RT-PCR and qRT-PCR analyses

Total RNAs from 200 mg of flowers, roots, stems, leaves and siliques (500 flowers collected for pollen) were collected from wild-type or mutant plants with RNA extract kit (TianGen, Product No. DP441, http://tiangen.casmart.com.cn, China) and treated with DNase I (Sigma, No.D5025, http://www.sigmaaldrich.com, USA) at 37 °C for 15 min. The first-strand cDNA was synthesized in a 20-μL reaction mixture containing 5 μg of total RNAs, oligo (dT) primers and M-MLV (TaKaRa, Product No. 2640A). The primer pairs *MTOPVIB*-A-F/*MTOPVIB*-A-R, *MTOPVIB*-B-F/*MTOPVIB*-B–R (Supplementary Table S2) were used to measure the expression levels of *MTOPVIB* in *mtopVIB*-*3* as described previously^50^. The primer pair of *MTOPVIB*-F/*MTOPVIB*-R (Supplementary Table S5) was used to examine the gene expression in the mutant *mtopVIB*-*2*. *TUBULIN8* (*AT5G23860*) and *AtSPO11*-*1* cDNA were used as internal references to normalize the amount of cDNA template using the primer pair *TUBULINrt*-F/*TUBULINrt*-R and *AtSPO11*-*1*-*F*/*AtSPO11*-*1*-*R* (Supplementary Table S5). qRT-PCR was performed as described previously^54^. The Power SYBR Green PCR master mix (Applied BioSystems, www.appliedbiosystem.com, USA) was used as described by the supplier’s instruction for the 7500 Real-time PCR system (Applied BioSystems). The qRT-PCR program was set as follows: 95 °C for 10 min, followed by 40 cycles of 95 °C for 15 s and 60 °C for 1 min. The gene-specific QRT-*MTOPVIB*-F, QRT-*MTOPVIB*-R and QRT-*AtSPO11*-*1*-F, QRT-*AtSPO11*-*1*-R primers used for qRT-PCR are listed in Supplementary Table S5. *ACTIN2* (*AT3G18780*) was used as the internal controls.

### Recombination frequency calculation

To measure the recombination frequencies, *mtopVIB*-*3* (L*er*) was used to cross with *mtopVIB*-*2*/+ (Col-0) for their polymorphisms between the L*er* and Col background. The F1 progeny of *mtopVIB*-*3*/+ as a control and *mtopVIB*-*2*/*mtopVIB*-*3* mutant plants were identified by their sterility and T-DNA insertions. The markers F3P11 and T16B24 on chromosome II and T26D22 and K6M13 on chromosome V were used for the assays. The selected plants were grown to maturity, and their seeds were germinated on MS agar plates. DNA was then isolated from the seedlings and used for genotyping by PCR as previously described^55^.

### Yeast two-hybrid and three-hybrid assays

The yeast two-hybrid (Y2H) assays were performed using the Gal4 vector system (Clontech, www.clontech.com). The CDSs of *MTOPVIB*, *AtSPO11*-*1*, *AtSPO11*-*2*, *AtPRD1*, *AtPRD2*, *AtPRD3* and *AtDFO* were cloned into both pGBKT7 vector and pGADT7 vector. The constructs were co-transformed into yeast strain AH109 as described previously^56^. The transformed cells were adjusted to OD_600_ = 0.4~0.6 and grown in SD/-Trp-Leu plates and SD/-Trp-Leu-His-Ade plates for 3 to 7 days at 30 °C. The yeast three-hybrid (Y3H) assays were performed using the pBridge vector system (Clontech, www.clontech.com). The CDSs of *MTOPVIB*, *AtSPO11*-*1*, *AtPRD1*, *AtPRD3* and *AtDFO* were cloned into pBridge vector, and the transformed cells were grown in SD/-Trp-Leu-Met plates and SD/-Trp-Leu-His-Met plates for 5 to 8 days at 30 °C. The primers used in the experiments are listed in Supplementary Table S6.

### Western blot analysis

Samples were separated on 8% SDS-PAGE gels and directly blotted onto PVDF membrane in a semi-dry chamber for 60 min at 100 V. After 2 h blocking with 5% defatted milk in TBST, the membrane was probed with either mouse anti-HA antibodies (Sigma, Product No. SAB2702196, dilution at 1/5000) for AD fusion or mouse anti-c-Myc (Sigma, Product No. M4439, dilution at 1/5000) for BD fusion. HRP-conjugated monoclonal anti-mouse antibodies (EasyBio, Product No. BE0107-100, Beijing, China) were used at a 1/5000 dilution. Secondary antibodies were revealed by chemiluminescence with ECL (Millipore, Product No. WBKLS0100, USA) and signals were examined using Fusion Solo system.

### Bimolecular fluorescence complementation assays

The coding sequences of the genes were amplified by PCR using the primers listed in Supplementary Table S7, and then cloned into the vectors pSPYNE-35S and pSPYCE-35S^57^. AtPRD1 was also cloned in pSUPER1300. The resulting constructs were introduced into onion epidermal cells using a biolistic PDS-1000/He gene gun system (Bio-Rad, http://www.bio-rad.com). Fluorescent signals were examined using a confocal laser scanning microscope (LSM710, Carl Zeiss, http://www.zeiss.com) 16–24 h after transformation.

## Electronic supplementary material

supplemental infomation
