# Supplementary material for: MTOPVIB interacts with AtPRD1 and plays important roles in formation of meiotic DNA double-strand breaks in Arabidopsis
Source: Sci Rep. 2017 Aug 30;7:10007. doi: 10.1038/s41598-017-10270-9 (PMC5577129; doi:10.1038/s41598-017-10270-9)
Supplement: Supplementary file 1 — supplemental infomation [file 41598_2017_10270_MOESM1_ESM.pdf]

MTOPVIB interacts with AtPRD1 and plays important roles in formation of meiotic DNA double-strand breaks in *Arabidopsis*

Yu Tang[1], Zhongnan Yin[1], Yuejuan Zeng[1], Qinxin Zhang[1], Liqun Chen[1], Yan He[2], Pingli Lu[3], De Ye[1] and Xueqin Zhang[1]*

[1]State Key Laboratory of Plant Physiology and Biochemistry, College of Biological Sciences, China Agricultural University, Beijing 100193, China

[2]College of Agriculture and Biotechnology, China Agricultural University, Beijing 100193, China

[3]School of Life Sciences, Fudan University, Shanghai 200433, China

*corresponding author: kouyang@cau.edu.cn

Print to PDF without this message by purchasing novaPDF (http://www.novapdf.com/)

Supplementary Information

Table S1. Genetic analysis of *mtopVIB-2* mutant.

Crosses (Female x Male)	Number of SD^R seedlings	Number of SD^S seedlings	SD^R/SD^S
mtopVIB-2/+ self-pollinated	1869	645	2.90
mtopVIB-2/+ × Wild-type	652	681	0.96
Wild-type × *mtopVIB-2*/+	1410	1449	0.97

SD^R, Sulfadiazine-resistant; SD^S, Sulfadiazine-sensitive.

Print to PDF without this message by purchasing novaPDF (http://www.novapdf.com/)

Table S2. The interactions of AtPRD1, MTOPVIB, AtSPO11-1, AtSPO11-2 and AtPRD3, AtDFO.

BD / AD	pGBKT7	MTOPVIB	AtSPO11-1	AtSPO11-2	AtSPO11-2 (1-210)	AtSPO11-2 (170-383)	AtPRD3	AtDFO	AtPRD1	AtPRD1 (1-821)	AtPRD1 (775-1330)
pGADT7	×	×	×	×	×	×	×	×	×	×	×
MTOPVIB	×	×	√√	×	√√	×	×	×	×	×	×
AtSPO11-1	×	√√	×	×	×	×	×	×	×	√√	×
AtSPO11-2	×	×	×	×	×	×	×	×	×	×	×
AtPRD3	×	×	×	×	×	×	√√	×	×	×	×
AtDFO	×	×	×	×	×	×	×	×	×	×	×
AtPRD1	×	√√	×	×	×	×	×	×	×	×	×
AtPRD1 (1-821)	×	√√	×	×	×	√√	√	√	√√	√√	×
AtPRD1 (775-1330)	×	×	×	×	×	×	×	×	×	×	×

The yeast two-hybrid assay showed that: (1) MTOPVIB interacted with AtSPO11-1; (2) MTOPVIB interacted with AtSPO11-12(1-210); (3) AtPRD1 interacted with MTOPVIB; (4) AtPRD1 (1-821 aa) interacted with AtSPO11-2 (170-383 aa); (5) AtPRD3 interacted with itself; (6) AtPRD1 (1-821 aa) interacted with AtPRD3 and AtDFO. (√√) growth well, (√) growth, (×) not growth on SD/-Leu-Trp-His-Ade medium.

Print to PDF without this message by purchasing novaPDF (http://www.novapdf.com/)

Table S3. The Y2H assay for the interaction of the truncated AtPRD1 proteins with Topo VI-like complex, AtPRD3 and AtDFO.

BD / AD	pGBKT7	MTOPVIB	MTOPVIB (1-261)	MTOPVIB (228-493)	AtSPO11-1	AtSPO11-2	AtSPO11-2 (1-210)	AtSPO11-2 (170-383)	AtPRD3	AtDFO	AtPRD1 (1-388)
pGADT7	×	×	×	×	×	×	×	×	×	×	×
PRD1-A(1-388)	×	√√	√√	×	√√	×	×	√√	√√	√	√√
PRD1-B(389-812)	×	×	×	×	×	×	×	×	×	×	×
PRD1-C(813-962)	×	×	×	×	×	×	×	×	×	×	×
PRD1-D(963-1109)	×	√	√	×	×	×	×	×	√	√	√
PRD1-E (1110-1330)	×	×	×	×	×	×	×	×	×	×	×

The yeast two-hybrid assay showed that truncated proteins, AtPRD1-A (1-388 aa) and AtPRD1-D (963-1109 aa) could interact with MTOPVIB, AtPRD3 and AtDFO in yeast. In addition, AtPRD1-A (1-388 aa) could interact with itself, and AtPRD1-A (1-388 aa) could interact with AtSPO11-1 and AtSPO11-2 (170-383 aa). (√√) growth well, (√) growth, (×) not growth on SD/-Leu-Trp-His-Ade medium.

Print to PDF without this message by purchasing novaPDF (http://www.novapdf.com/)

Table S4. Number of cells showing GFP signal of BiFC assay for the interaction of MTOPVIB with AtSPO11-1 and AtSPO11-2, AtPRD1 with Topo VI-like complex, AtPRD3 and AtDFO.

Conbination of interactions	Number of cells showing GFP signal
p35S::*GFP*	41
MTOPVIB + AtSPO11-1	12
MTOPVIB + AtSPO11-2	13
AtPRD1 + AtSPO11-1	5
AtPRD1 + AtSPO11-2	5
AtPRD1 + MTOPVIB	5
AtPRD1 + AtPRD3	1
AtPRD1 + AtDFO	2
AtPRD1-A (1-388) + AtSPO11-1	5
AtPRD1-A (1-388) + AtSPO11-2	7
AtPRD1-A (1-388) + MTOPVIB	2
AtPRD1-A (1-388) + AtPRD3	6
AtPRD1-A (1-388) + AtDFO	2
AtPRD1-A (963-1109) + MTOPVIB	1
AtPRD1-A (963-1109) + AtPRD3	1
AtPRD1-A (963-1109) + AtDFO	2
AtDFO+AtPRD3	0
AtPRD1+AtDFO+AtPRD3	3

The strength order of interactions: 1. MTOPVIB with AtSPO11-1 and AtSPO11-2; 2. AtPRD1 with Topo VI-like complex; 3. AtPRD1 with AtPRD3 and AtDFO. The nagtive control in the interaction of MTOPVIB with AtSPO11-1 and AtSPO11-2, AtPRD1 with Topo VI-like complex, AtPRD3 and AtDFO showed no GFP signals. *p35S*::*GFP* was used as the positive control.

Print to PDF without this message by purchasing novaPDF (http://www.novapdf.com/)

Table S5. The sequences of the primers used in this study.

Uses	Primers	Sequences (5'->3')
Identification of T-DNA insertion sites	mtopVIB-2-F	AGTGACACAGTGCTTGAGGA
	mtopVIB-2-R	CCAGAAGGCCAACAATACCA
	Atmre11-F	AGTGGGAGAGTGCTTAGAGGC
	Atmre11-R	TACCACGTTTTGAAGTCCCAG
	Atcom1-1-F	GGTTGAGGAAGGAAAGACAGC
	Atcom1-1-R	GCGTATCTGTAACGATGCCTC
	Atrad50-1-F	TCAAAAACTGCCCGTAGTTTG
	Atrad50-1-R	ACCTCAGTTTGAAATCCCGAG
	Atrad51-F	TTCAGGATGGTGTCTCAGAGC
	Atrad51-R	ATGCCAAGGTTGACAAGATTG
	Wis-p745	AACGTCCGCAATGTGTTATTAAGTTGTC
	SAIL-LB3	TAGCATCTGAATTTCATAACCAATCTCGATACAC
	SALK_LBa1	TGGTTCACGTAGTGGGCCATCG
	GK-LB	ATATTGACCATCATACTCATTGC
Genetic mapping	F2J6-F	GCTGCTCAAAATATTCTTTA
	F2J6-R	CTCAGCGTGAGTCTAGAT
	T6C23-F	CTTGCCTTACGATACATTTG
	T6C23-R	TGCAGCATTTTATCTAATCC
	T30E16-F	AGATGCAAATTCCAAGAAAC
	T30E16-R	GCCAGACATACTTTCATGTG
	T13D8-F	GTTAGCTCTTCCGAGATCTG
	T13D8-R	CGGCGAGAATGATGGAAGGC
	F8A5-F	GCTCTGTTAGGTACGCCTTTTGTTACAAAC
	F8A5-R	GTGAGTAACGTGCATGTTGTTGGAATC
	F23C21-F	TTTGATTTATAACGAGAGAT
	F23C21-R	ATTGCACATATTTCTGTTTT
	T7P1-F	ATGTACCATCTTACCATTAT
	T7P1-R	GACCATTGGATTGCTAAC
	F11P17-F	TATGCATGCTTCTCTGCATAATGA
	F11P17-R	TGCTGATGCTTTCGGGGCTTCAAG
	F8K4-F	GCTATTATTGTGGTCGTATT
	F8K4-R	TTGTTACATGCTTTAAGATT
Complementation and conformation of the transgenes	MTOPVIB-COM-F（KpnI）	GGGGTACCTGCGCCAAAGGAAAATGAAGA
	MTOPVIB-COM-R（SalI）	GCGTCGACCTTGCAGGGAAGTCACAAGA
	YZ-MTOPVIB-F	ACCGGTGTTGTTGAAAGGAA
	R-1300-HindIII	TGCAAGGCGATTAAGTTGGG
	F-1300-EcoRI	CTTCCGGCTCGTATGTTGTG
	YZ-MTOPVIB-R	ACTGGCTTTGGACAAACTAGC
RT-PCR and Quantitative RT-PCR	RT-M TOPVIB-A-F	AGTGACACAGTGCTTGAGGA
	RT-MTOPVIB-A-R	AAGCCAATTATCGGTGCCAC
	RT-MTOPVIB-B-F	AGTCAACTCAGCATTGCAGC
	RT-MTOPVIB-B-R	AGGAGCAGCTTCTTGGTCTT
	RT-AtSPO11-1-F	CGGTCGTTGTAGATCTGGCT
	RT-AtSPO11-1-R	TAACAATGCAGCGGTTCGTC
	RT-mtopVIB-2-F	TTGGGATGGGTTACTCTCCG
	RT-mtopVIB-2-R	CTTCCATCGTCCCTCCAACT
	F-Tubulin	CTTCGTATTTGGTCAATCCGGTGC
	R-Tubulin	GAACATGGCTGAGGCTGTCAAGTA
	QRT-MTOPVIB-F	AGTGACACAGTGCTTGAGGA
	QRT-MTOPVIB-R	CGGAGAGTAACCCATCCCAA
	QRT-AtSPO11-1-F	TGCAGCTCTGATAAACCAAAGG
	QRT-AtSPO11-1-R	CCAATTGCACGGTCCACAAT
	ACTIN2-F	GGTAACATTGTGCTCAGTGGTGG
	ACTIN2-R	AACGACCTTAATCTTCATGCTGC
Measurement of recombination frequencies	F3P11-F	ATGTATTTGTTGCAAAATAA
	F3P11-R	TGCACAGAAGAAAAAACTA
	T16B24-F	ATGAACGGAGTAGCTATC
	T16B24-R	CGCGTAGAACATAATCTGTA
	T26D22-F	CACAGGCCATTGGATGTA
	T26D22-R	TGTTAGAACCCACCATTTG
	K6M13-F	CCTGTTCCAATGAATATG
	K6M13-R	TGTAGCTGCTGAGTTGTC

-F, forward primer; -R, reverse primer.

Print to PDF without this message by purchasing novaPDF (http://www.novapdf.com/)

Table S6. The sequences of the primers used in Y2H and Y3H assays.

Uses for	Primers	Sequences (5'->3')
PRD3	Y2H-PRD3-F (NdeI)	GGAATTCCATATG ATGAAGATGAATATTAACAAAGCC
	Y2H-PRD3-F (BamHI)	CGGGATCC GTTAATTATTATGGGGTTACCG
	Y3H-SPO11-1-F (BamHI)	CGGGATCCGT ATGGAGGGAAAATTCGCTATTTCAG
	Y3H-SPO11-1-R (PstI)	AACTGCAG TCATCAAGGAGAGCTTACTTCACGAC
PRD2	Y2H-PRD2-F (NdeI)	GGAATTCCATATG ATGAGTTCAAGCGTAGCTGAAG
	Y2H-PRD2-F (EcoRI)	GGAATTC TCATTCTATTTCTTGGTAGGCTAAG
PRD1	Y2H-PRD1-1-F (XmaI)	TCCCCCCGGG ATGTTCTTCCAACACTCACAGTTG
	Y2H-PRD1-1-R（BamHI）	CGGGATCC CATGGATAAATTGTTGGAGGC
	Y2H-PRD1-2-F（SfiI）	ATGGCCATGGAGGCC AACTATGCCGCAACTCTTCTAGTC
	Y2H-PRD1-2-R (BamHI)	CGGGATCC CTACACGATTCTCTCTGTTTGCA
SPO11-1	Y2H-SPO11-1-F (EcoRI)	GGAATTC ATGGAGGGAAAATTCGCTATTTCAG
	Y2H-SPO11-1-R (BamHI)	CGGGATCC TCAAGGAGAGCTTACTTCACGAC
	Y3H-SPO11-1-F (BamHI)	CGGGATCCGT ATGGAGGGAAAATTCGCTATTTCAG
	Y3H-SPO11-1-R (PstI)	AACTGCAG TCATCAAGGAGAGCTTACTTCACGAC
SPO11-2	Y2H-SPO11-2-1-F (EcoRI)	GGAATTC ATGGAGGAAAGTTCAGGACTATC
	Y2H-SPO11-2-1-R (BamHI)	CGGGATCC TCAAGCATCAGTTCTCATGATGGT
	Y2H-SPO11-2-2-F (EcoRI)	GGAATTC GGAAGGTTATTTCTACAAGAAC
	Y2H-SPO11-2-2-R (BamHI)	CGGGATCC TATGTATTTGCCTTGCACGATCTT
MTOPVIB	Y2H-MTOPVIB-1-F（SfiI）	ATGGCCATGGAGGCC ATGGAAAACAATGCTCCGGTTC
	Y2H-MTOPVIB-1-R（BamHI）	CGGGATCC TATCACAATAACCACTTCCATCG
	Y2H-MTOPVIB-2-F（SfiI）	ATGGCCATGGAGGCC GTTGGAGGGACGATGGAAG
	Y2H-MTOPVIB-2-R (BamHI)	CGGGATCC CTATTCCTGCAGCATAGTCGCC
	Y3H-MTOPVIB-F (BamHI)	CGGGATCCGT ATGGAAAACAATGCTCCGGTTC
	Y3H-MTOPVIB-R (SalI)	ACGCGTCGAC CTATTCCTGCAGCATAGTCGCC
DFO	Y2H-DFO-F (SfiI)	ATGGCCATGGAGGCCATGCGCCATAACATAAAATTCAAATC
	Y2H-DFO-R (BamHI)	CGGGATCC AAATGTAGTCAGAAGTCTGTTACAAAC
	Y2H-DFO-F (NdeI)	GGAATTCCATATG ATGCGCCATAACATAAAATTCAAATC
	Y2H-DFO-R (PstI)	AACTGCAG AAATGTAGTCAGAAGTCTGTTACAAAC
	Y3H-DFO-F (BamHI)	CGGGATCCGT ATGCGCCATAACATAAAATTCAAATC
	Y3H-DFO-R (SalI)	ACGCGTCGAC TCAAAATGTAGTCAGAAGTCTGTTACAAAC
PRD1	Y2H-PRD1-1-F (XmaI)	TCCCCCCGGG ATGTTCTTCCAACACTCACAGTTG
	Y2H-PRD1-1-R（BamHI）	CGGGATCC CATGGATAAATTGTTGGAGGC
	Y2H-PRD1-2-F（SfiI）	ATGGCCATGGAGGCC AACTATGCCGCAACTCTTCTAGTC
	Y2H-PRD1-2-R (BamHI)	CGGGATCC CTACACGATTCTCTCTGTTTGCA
	Y2H-A-PRD1-F (XmaI)	TCCCCCCGGG ATGTTCTTCCAACACTCACAGTTG
	Y2H-A-PRD1-R（BamHI）	CGGGATCC CCCAATAACAAGTCTCTTCC
	Y2H-B-PRD1-F (SfiI)	ATGGCCATGGAGGCC TTTCCTTCAGTCATCCGAGTGC
	Y2H-B-PRD1-R（BamHI）	CGGGATCC CAACTTTGCAAACCAATATGTG
	Y2H-C-PRD1-F（NdeI）	GGAATTCCATATG ATATCAGAAGGAGACAACTATGC
	Y2H-C-PRD1-R (BamHI)	CGGGATCC AATATTTCTTGAAGCTTCCACA
	Y2H-D-PRD1-F（NdeI）	GGAATTCCATATG GTGTCGAATTCGTACCTGGTATC
	Y2H-D-PRD1-R (BamHI)	CGGGATCC AGAGTTTGTCGCTACTCTTATGTC
	Y2H-E-PRD1-F（NdeI）	GGAATTCCATATG GCGCTTTGTCTTTCCATGATTTTAG
	Y2H-E-PRD1-R (BamHI)	CGGGATCC CTACACGATTCTCTCTGTTTGCA
	Y3H-PRD1-A-F (NotI）	ATAAGAATGCGGCCGC AATGTTCTTCCAACACTCACAG
	Y3H-PRD1-A-R (BglII)	GAAGATCT CTACCCAATAACAAGTCTCTTCC

-F, forward primer; -R, reverse primer.

Print to PDF without this message by purchasing novaPDF (http://www.novapdf.com/)

Table S7. The sequences of the primers used in BiFC assays.

Uses	Primers	Sequences (5'->3')
MTOPVIB	BiFC-MTOPVIB-F (XbaI)	GCTCTAGAATGGAAAACAATGCTCCGGTTC
	BiFC-MTOPVIB-R (BamHI)	CGGGATCCTTCCTGCAGCATAGTCGCCAGAC
SPO11-1	BiFC-SPO11-1-F (XbaI)	GCTCTAGAATGGAGGGAAAATTCGCTATTTCAG
	BiFC-SPO11-1-R (BamHI)	CGGGATCCAGGAGAGCTTACTTCACGAC
SPO11-2	BiFC-SPO11-2-F (XbaI)	GCTCTAGAATGGAGGAAAGTTCAGGACTATC
	BiFC-SPO11-2-R (BamHI)	CGGGATCCTATGTATTTGCCTTGCACGATCTT
PRD3	BiFC-PRD3-F(BamHI)	CGGGATCCATGAAGATGAATATTAACAAAGCC
	BiFC-PRD3-R (KpnI)	GGGGTACCAATTATTATGGGGTTACCG
DFO	BiFC-DFO-F (BamHI)	CGGGATCCATGCGCCATAACATAAAATTCAAATC
	BiFC-DFO-R (KpnI)	GGGGTACCAAATGTAGTCAGAAGTCTGTTACAAAC
PRD3	BiFC-PRD3-F (XbaI)	GCTCTAGAATGAAGATGAATATTAACAAAGCC
	BiFC-PRD3-R (BamHI)	CGGGATCCGTTAATTATTATGGGGTTACCG
PRD1	BiFC-PRD1-F (BamHI)	CGGGATCC ATGTTCTTCCAACACTCACAGTTG
	BiFC-PRD1-R (KpnI)	GGGGTACCCACGATTCTCTCTGTTTGCA
	BiFC-PRD1-A-F (BamHI)	CGGGATCCATGTTCTTCCAACACTCACAGTTG
	BiFC-PRD1-A-R (KpnI)	GGGGTACCCCCAATAACAAGTCTCTTCC
	BiFC-PRD1-D-F (BamHI)	CGGGATCCGTGTCGAATTCGTACCTGGTATC
	BiFC-PRD1-D-R (KpnI)	GGGGTACCAGAGTTTGTCGCTACTCTTATGTC
	SUPER-AtPRD1-F (ApaI)	ATGGGGGCCC ATGTTCTTCCAACACTCACAGTTG
	SUPER-AtPRD1-R (SpeI)	GACTAGT TGCAAACAGAGAGAATCGTG
MTOPVIB	BiFC-MTOPVIB-F (XbaI)	GCTCTAGAATGGAAAACAATGCTCCGGTTC
	BiFC-MTOPVIB-R (BamHI)	CGGGATCCTTCCTGCAGCATAGTCGCCAGAC
	BiFC-MTOPVIB-1-F (KpnI)	GGGGTACCATGGAAAACAATGCTCCGGTTC
	BiFC-MTOPVIB-1-R (BamHI)	CGGGATCC GCTGCAATGCTGAGTTGACT
	BiFC-MTOPVIB-2-F (KpnI)	GGGGTACCGGAACAGTATGCCAGGAAGA
	BiFC-MTOPVIB-2-R (BamHI)	CGGGATCCTTCCTGCAGCATAGTCGCC

-F, forward primer; -R, reverse primer.

Print to PDF without this message by purchasing novaPDF (http://www.novapdf.com/)

Figure S1.

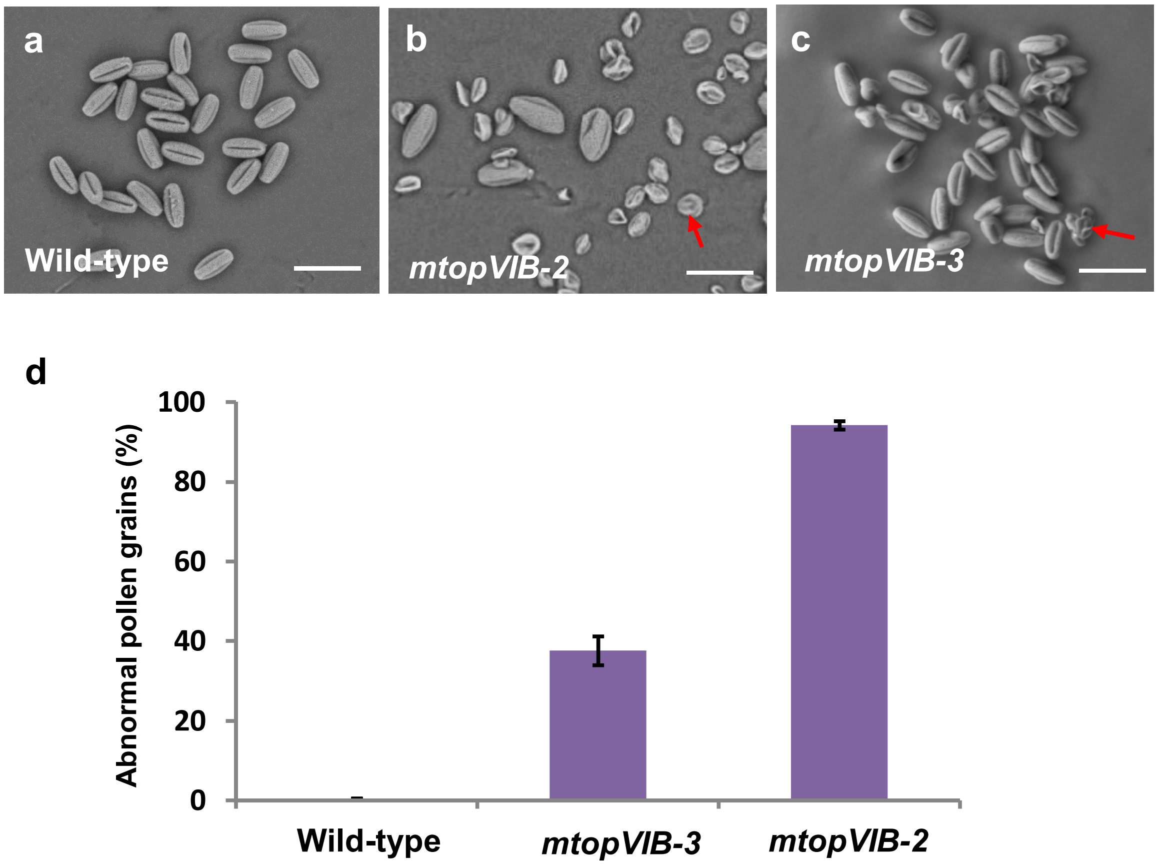

Figure S1. Defects of pollen grains in *mtopVIB-2* and *mtopVIB-3* mutants.

(a-c) SEM images of the pollen grains from wild type (a), *mtopVIB-2* (b), and *mtopVIB-3* (c). (d) A quantitative comparison of the abnormal pollen grains in wild-type (L*er*), *mtopVIB-2* and *mtopVIB-3*. The red arrows indicate the abnormal pollen grains. Bars=50 µm in (a-c).

Print to PDF without this message by purchasing novaPDF (http://www.novapdf.com/)

Figure S2.

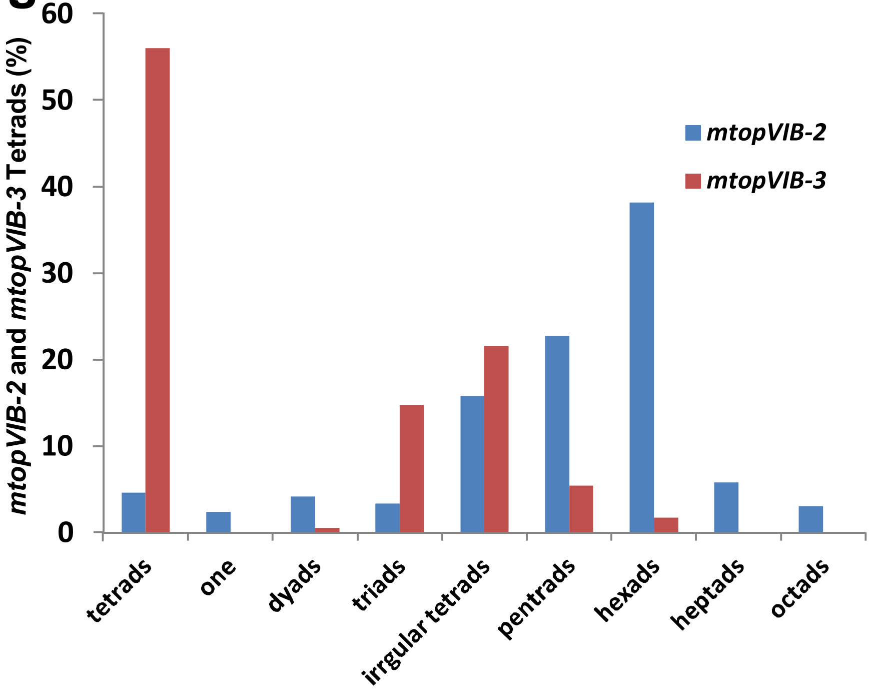

Figure S2. Quantitative comparison of the polyads in *mtopVIB-2* and *mtopVIB-3*.

mtopVIB-2 had 4.6% normal tetrads, 2.4% one microspore, 4.2% dyads, 3.3% triads, 15.8% irregular tetrads, 22.7% pentrads, 38.2% hexads, 5.8% heptrads and 3% octads. *mtopVIB-3* had 56% normal tetrads, 0.6% dyads, 14.8% triads, 21.6% irregular tetrads, 5.4% pentrads and 1.7% hexads.

Print to PDF without this message by purchasing novaPDF (http://www.novapdf.com/)

Figure S3.

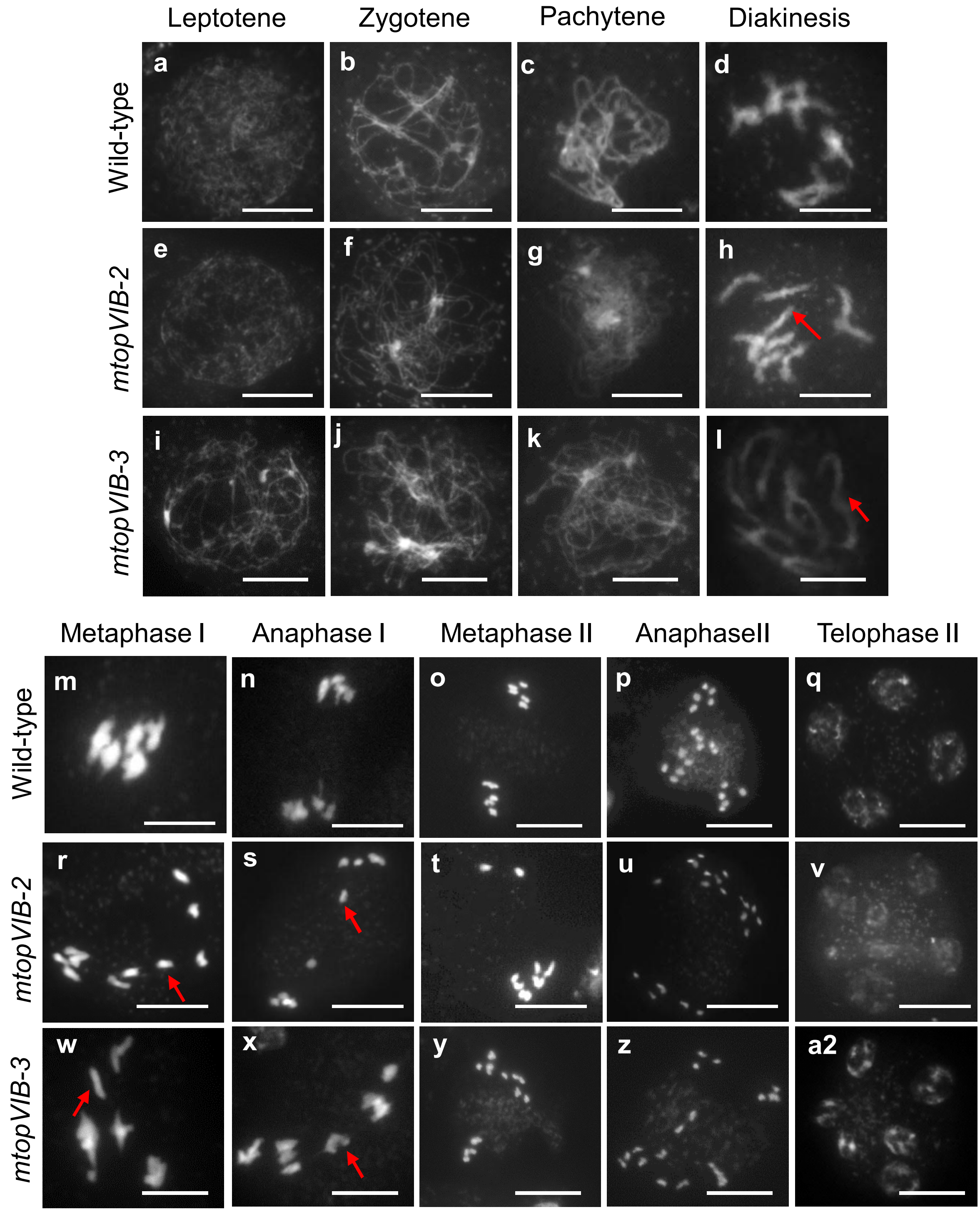

Figure S3. Aberrant meiosis in male meiocytes from *mtopVIB-2* and *mtopVIB-3*. (a-d) Meiotic chromosome behaviors at the early meiotic stages in wild-type (WT) plant. (e-h) Meiotic chromosome behaviors at the early meiotic stages in *mtopVIB-2.* (i-l) Meiotic chromosome behaviors at the early meiotic stages in *mtopVIB-3*. (m-q) Meiotic chromosome behaviors at the middle and late stages in wild-type plant. (r-v) Meiotic chromosome behaviors at the middle and late stages in *mtopVIB-2.* (w-a2) Meiotic chromosome behaviors at the middle and late meiotic stages in *mtopVIB-3.* The univalents at diakinesis stage and Metaphase I and lagging chromosomes at anaphase I in *mtopVIB* mutants are indicated by red arrows. Bars=10 μm.

Print to PDF without this message by purchasing novaPDF (http://www.novapdf.com/)

Figure S4.

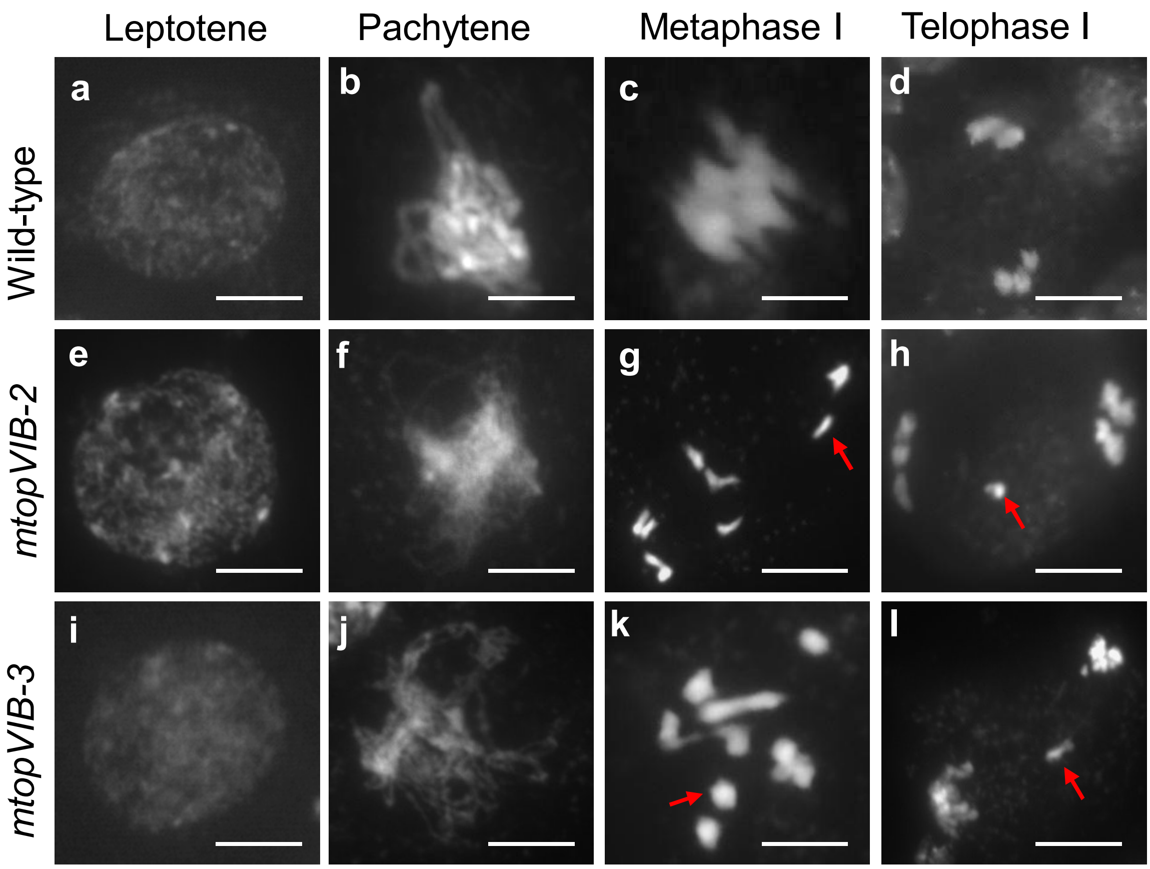

Figure S4. Aberrant meiosis in female meiocytes from *mtopVIB-2* and *mtopVIB-3*. Meiotic chromosomes at the different stages in wild-type (a-d), *mtopVIB-2* (e-h), *mtopVIB-3* (i-k). The red arrows indicate the univalents at metaphase I and lagging chromosomes at telophase I in the *mtopVIB* mutants. Bars=10 μm.

Print to PDF without this message by purchasing novaPDF (http://www.novapdf.com/)

Figure S5.

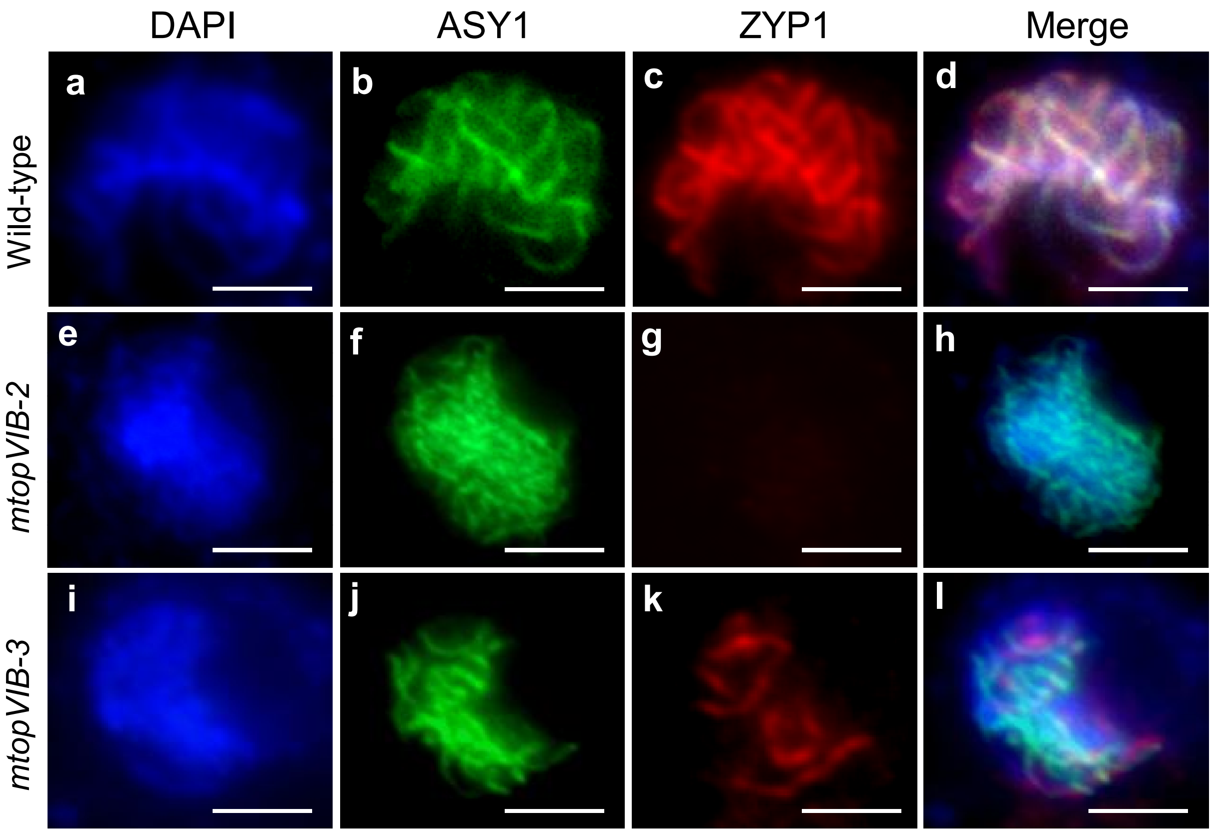

Figure S5. Co-immunolocalization assays of ASY1 and ZYP1 in wild-type, *mtopVIB-2* and *mtopVIB-3* meiocytes at the pachytene stages.

Co-immunolocalization of ASY1 (green) and ZYP1 (red) in the meiocytes at pachytenes in wild-type (a-d), *mtopVIB-2* (e-h) and *mtopVIB-3* (i-l). No ZYP1 signals in *mtopVIB-2* and weak ZYP1 signals in *mtopVIB-3* were observed. Bars=10 μm.

Print to PDF without this message by purchasing novaPDF (http://www.novapdf.com/)

Figure S6.

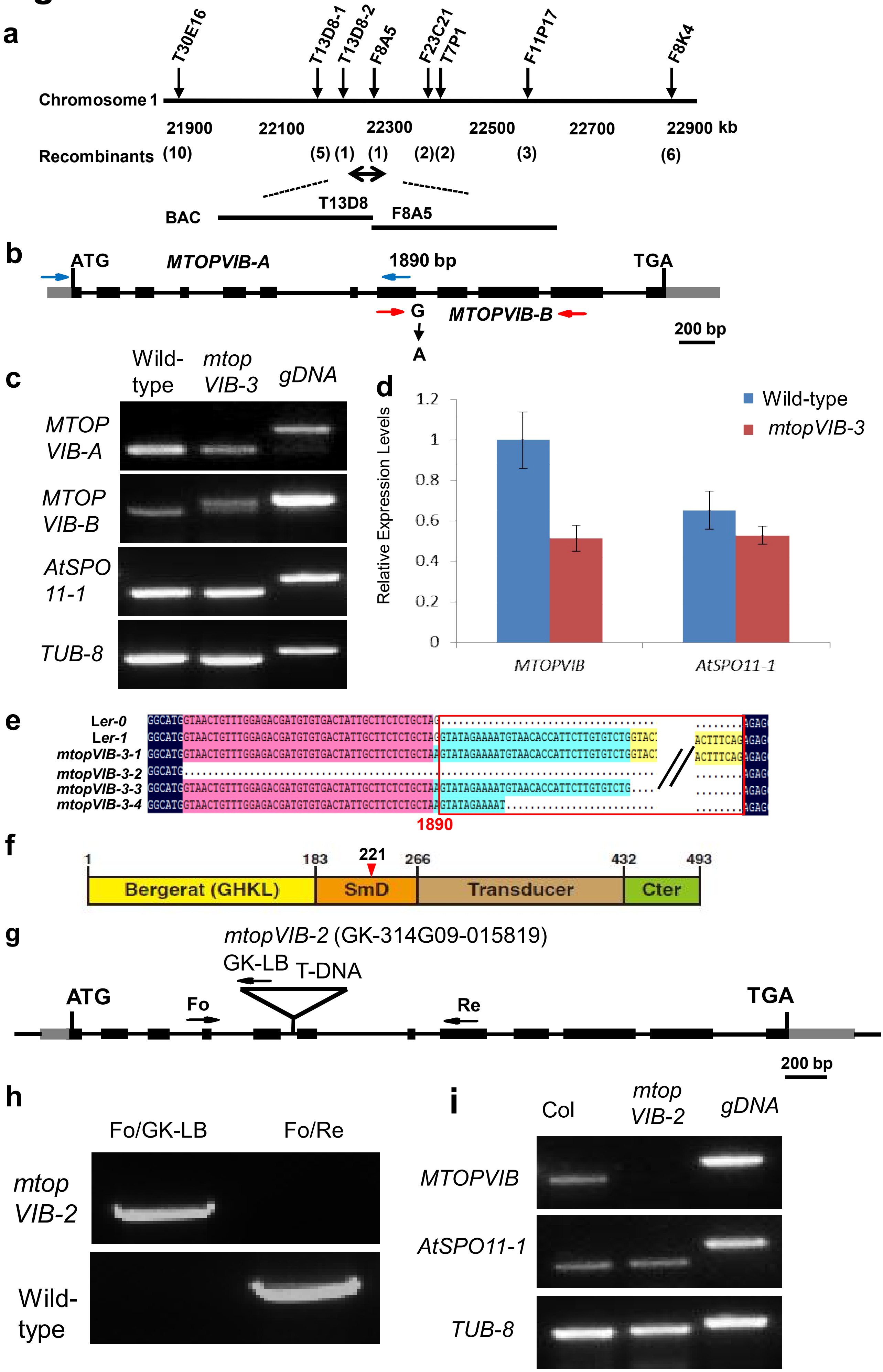

Print to PDF without this message by purchasing novaPDF (http://www.novapdf.com/)

Figure S6. Molecular characterization of the *mtopVIB* mutants.

(a) The *mtopVIB-3* mutation was located between the BAC clones T13D8 and F8A5 on chromosome 1. (b) Sequencing revealed that *mtopVIB-3* was caused by a G-to-A mutation at the position 1890 bp downstream of the start codon ATG in *AT1G60460* (*MTOPVIB*). The two blue arrows indicated the locations of the primers used for amplification of *MTOPVIB-A* fragment. The two red arrows indicated the locations of the primers for amplification of *MTOPVIB-B* fragment. (c) A RT-PCR assay for *MTOPVIB* expression in wild-type and *mtopVIB-3* inflorescences. *AtSPO11-1 and TUBULIN8* (*TUB-8*) were used as internal controls. (d) Relative expression levels of *MTOPVIB* in wild-type and *mtopVIB-3* plants, *ACTIN* and *AtSPO11-1* were used as internal controls. (e) Sequence alignment comparison of the *MTOPVIB* cDNA from L*er* and *mtopVIB-3* using DNAMAN. L*er-0* presented the mature product of *MTOPVIB* transcript. L*er-1* presented the intermediate product of the *MTOPVIB* transcript. The *mtopVIB-3-1* showed that the intron behind the mutation site was remained. The *mtopVIB-3-2* had a 43 bp deletion at the position upstream of the mutation site. The *mtopVIB-3-3* and *mtopVIB-3-4* showed 32 bp- and 11 bp-remains of the intron behind the mutation, respectively. Red box indicates the position of the eight intron. The slash lines presented the omitted/abbreviated sequences of the eight intron. (f) The *mtopVIB-3* mutation led to truncation of the protein and mutation at the site of 221 aa. (g) T-DNA was inserted in the fifth intron in *mtopVIB-2* mutant. (h) Confirmation of the T-DNA insertion in *mtopVIB-2* by PCR. (i) RT-PCR assay for expression of *MTOPVIB* in wild-type (WT) and *mtopVIB-2* inflorescences. Fo and Re presented the forward and reverse gene-specific primers for T-DNA insertion and RT-PCR assays. GK-LB presented the T-DNA left border (3'-end)-specific primer. WT, wild-type.

Print to PDF without this message by purchasing novaPDF (http://www.novapdf.com/)

Figure S7.

a

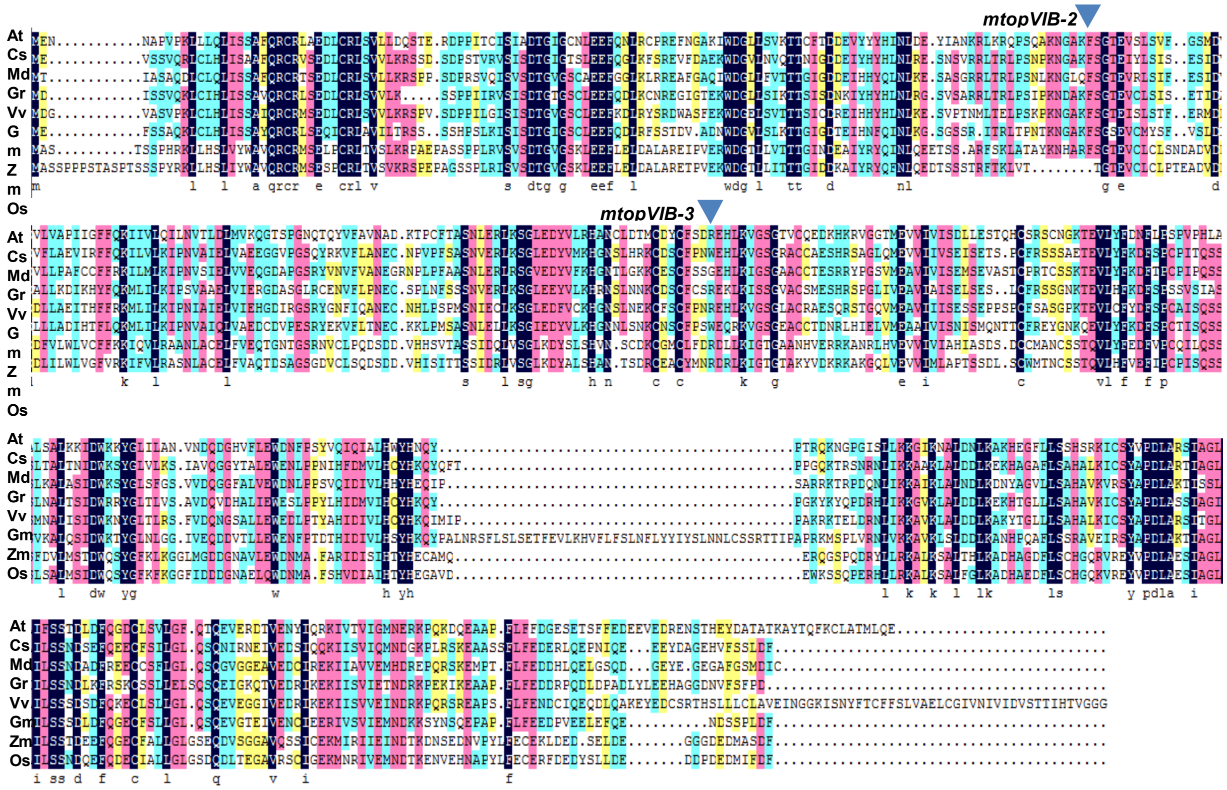

b

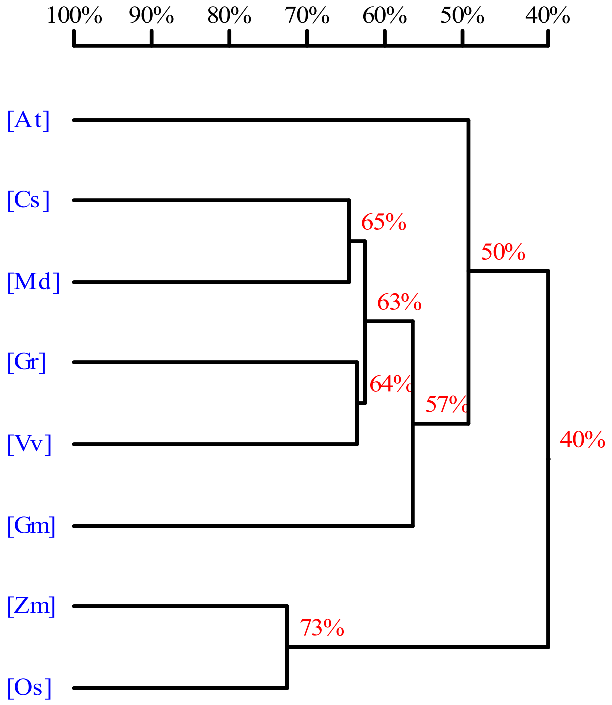

Print to PDF without this message by purchasing novaPDF (http://www.novapdf.com/)

Figure S7. Multiple sequence alignment of MTOPVIB and its homologs in plants.

(a) MTOPVIB was aligned with the seven MTOPVIB homologs from other species. The arrows indicated the mutation sites in *mtopVIB-2* and *mtopVIB-3*. (b) A philogenetic tree of MTOPVIB and the homologs in plants. At, *Arabidopsis thaliana* (MTOPVIB); Cs, *Citrus sinensis,* XP_006484319.1*;* Gm, *Glycine max* (XP_006607066.1); Gr, *Gossypium raimondii* (KJB65545.1); Md, *Malus domestica* (P_008353023.1); Mt, *Cucumis melo* (XP_008444327.1); Os, Oryza sativa (EEE66334.1); Vv, *Vitis vinifera* (XP_010651436.1); Zm, *Zea mays* (XP_008645058.1).

Print to PDF without this message by purchasing novaPDF (http://www.novapdf.com/)

Figure S8.

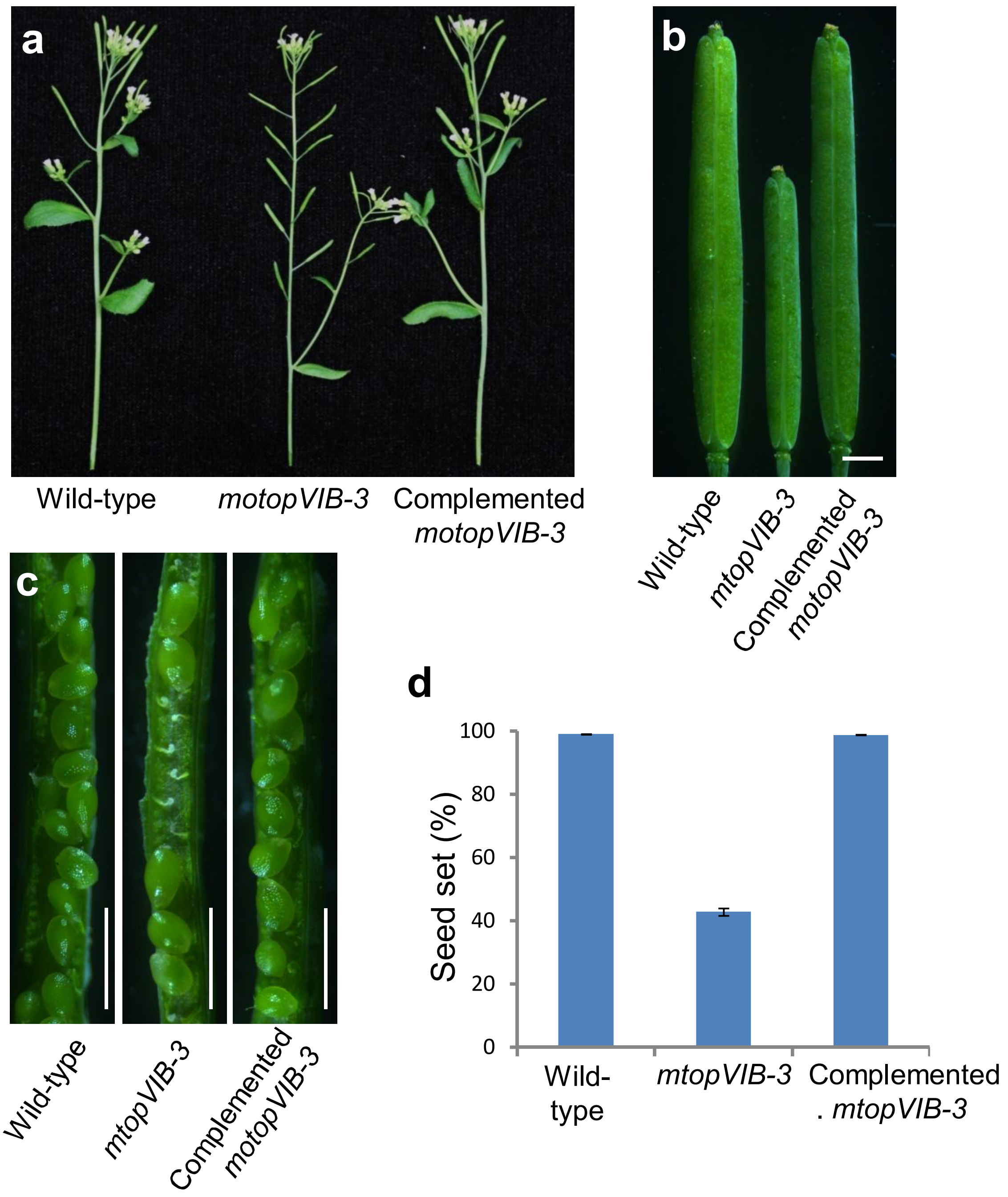

Figure S8. Complementation of the *mtopVIB-3* mutant phenotype.

(a) The inflorescences from wild-type, *mtopVIB-3* and complemented *mtopVIB-3* plants. (b) The siliques from wild-type, *mtopVIB-3* and complemented *mtopVIB-3* plants. (c) The seed sets siliques from wild-type, *mtopVIB-3* and complemented *mtopVIB-3* plants. (d) A quantitative comparison of seed-set rates in the siliques from wild-type, *mtopVIB-3* and complemented *mtopVIB-3* plants. Bars=2 mm.

Print to PDF without this message by purchasing novaPDF (http://www.novapdf.com/)

Figure S9.

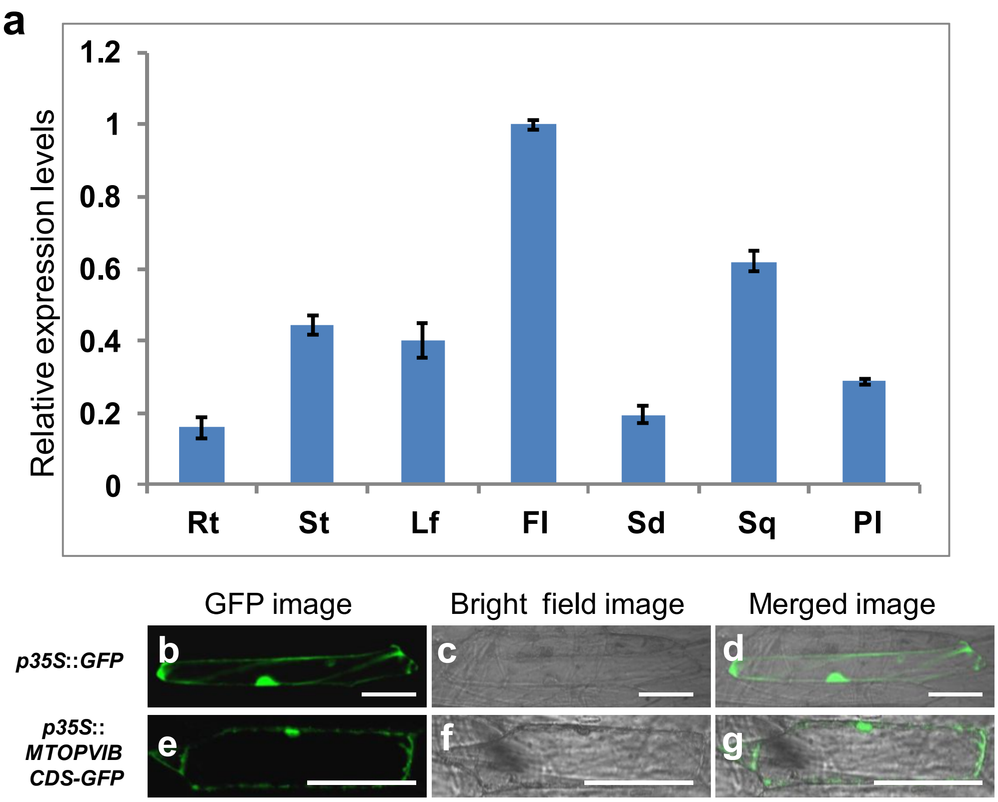

Figure S9. The characterization of *MTOPVIB*.

(a) A qRT-PCR assay for the relative expression levels of *MTOPVIB* in different tissues from wild-type plants. (b-l) Transient expression of green fluorescent protein (GFP) under control of 35S promoter in an onion epidermal cell was used as a control, showing that the GFP protein was distributed in the whole cell. (e-g) Transient expression of GFP-MTOPOVIB under 35S promoter in an onion epidermal cell, showing that the MTOPOVIB was localized in the nucleus and cytoplasm. Fl, flower; Lf, leaf; Pl, pollen; Rt, root; Sd, seedling; Sq, silique; St, stem. Bars=1 00 μm.

Print to PDF without this message by purchasing novaPDF (http://www.novapdf.com/)

Figure S10.

a

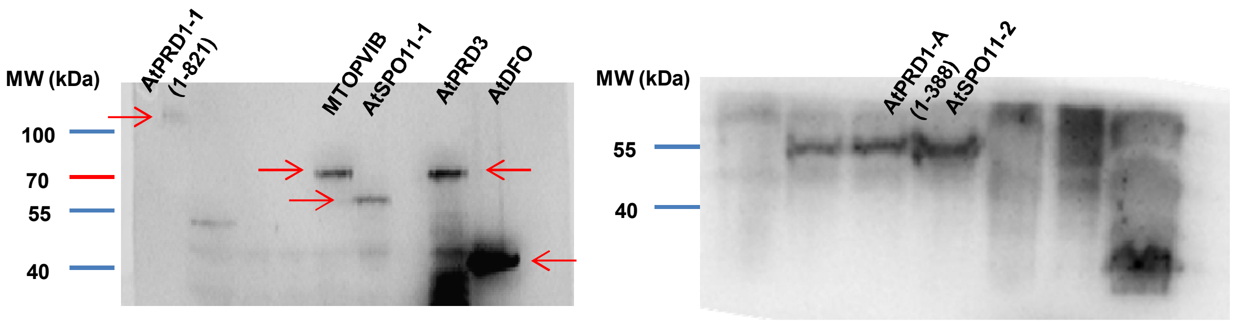

α-c-MYC (BD constructs)

b

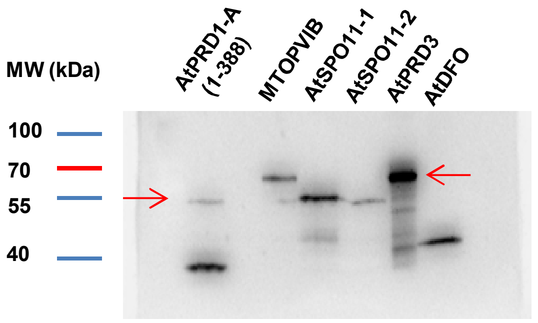

α-HA (AD constructs)

Figure S10. Western blot analysis of recombinant protein expression in yeast cells. Expression of MTOPVIB, SPO11-1, SPO11-2, AtPRD1, AtPRD3 and AtDFO was examined by western blot. BD recombinant proteins with anti-c-Myc antibody (a) and AD recombinant proteins were detected with anti-HA antibody (b). BD recombinant proteins and AD recombinant proteins were showed the full-length blot in (a) and (b), respectively.

AtPRD1-1 (1-821)=107 kDa; AtPRD1-A (1-388)=56 kDa; MTOPVIB=70 kDa; AtPRD3=69 kDa; AtSPO11-1=55 kDa; AtSPO11-2=56 kDa; AtDFO=40 kDa. Red arrows pointed in lanes are the target proteins. MW: Prestained Protein Ladder.

Print to PDF without this message by purchasing novaPDF (http://www.novapdf.com/)

Figure S11.

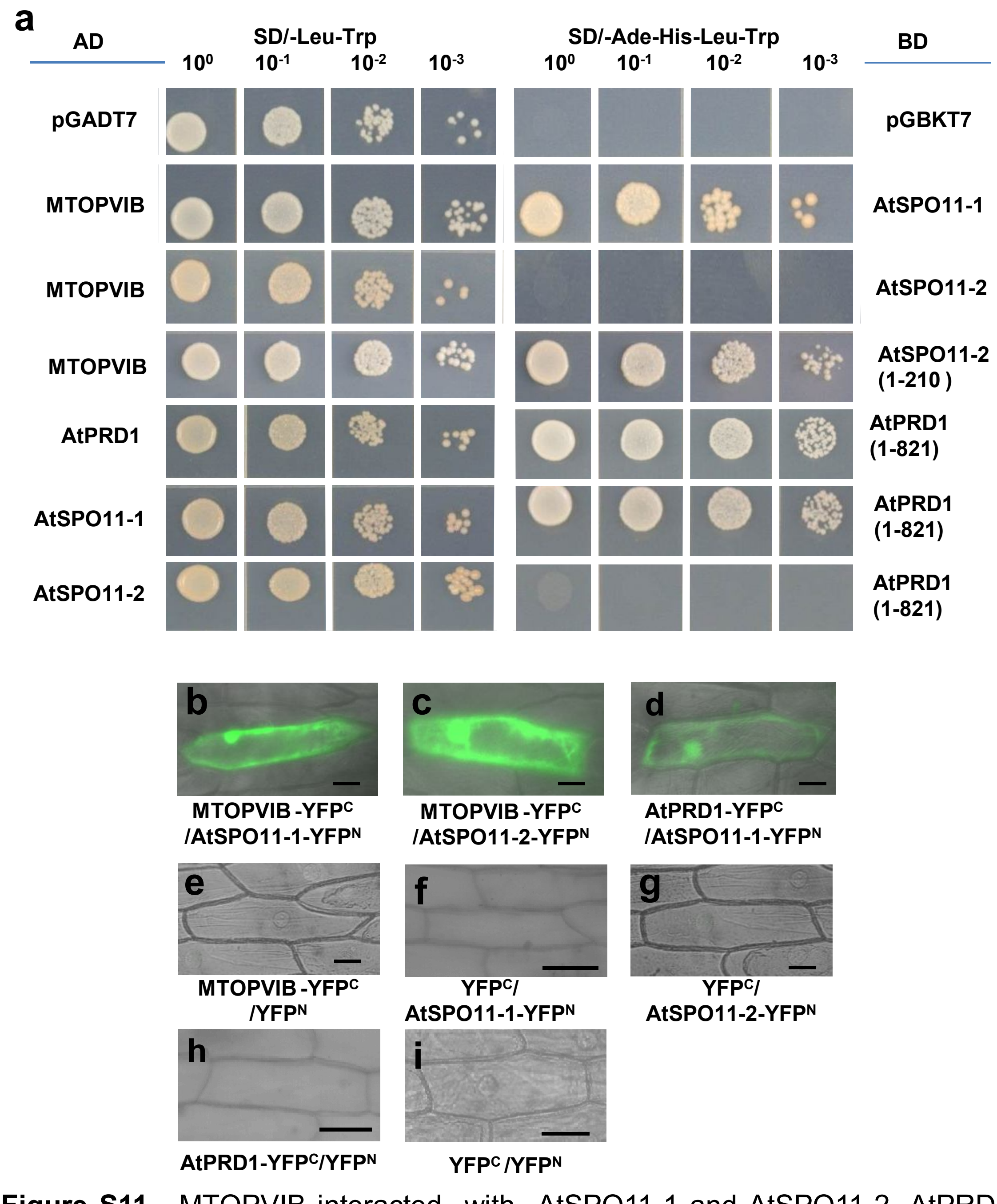

Figure S11. MTOPVIB interacted with AtSPO11-1 and AtSPO11-2, AtPRD1 interacted with AtSPO11-1 .

(a) The yeast two-hybrid assay showed that MTOPVIB interacted with AtSPO11-1 and AtSPO11-2 (1-210 aa), AtPRD1 (1-821 aa) interacted with AtSPO11-1 and itself. (b-i) Bimolecular fluorescence complementation assays showed interactions of MTOPVIB with AtSPO11-1 (b) and AtSPO11-2 (c), AtPRD1 with AtSPO11-1 (d) in onion epidermal cells. (e-i) The negative controls for (b-d). Bars=50 μm.

Print to PDF without this message by purchasing novaPDF (http://www.novapdf.com/)

Figure S12.

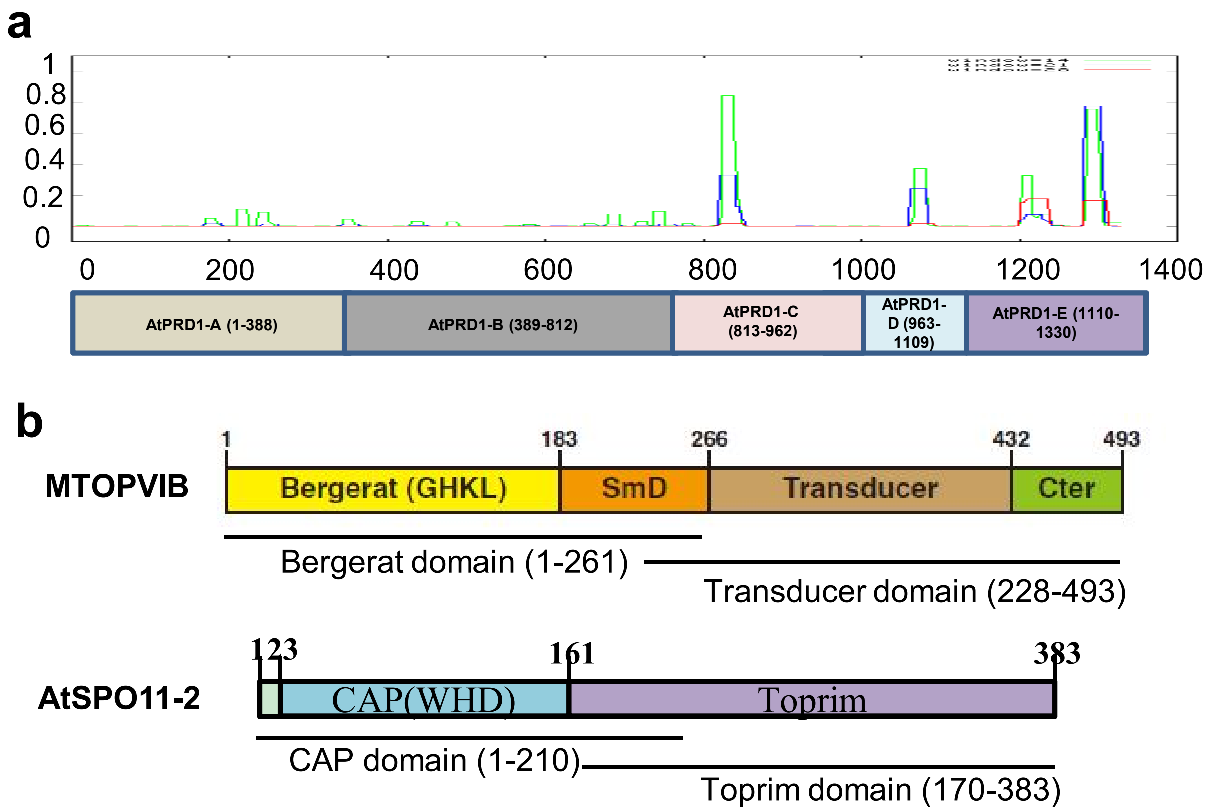

Figure S12. The structural characteristics of AtPRD1, MTOPVIB and AtSPO11-2 proteins.

(a) The expasy analysis (http://embnet.vital-it.ch/cgi-bin/COILS) showed that AtPRD1 had putative coiled-coils domains. For the further interaction assays, the AtPRD1 was divided into five regions: AtPRD1-A (1-388 aa), AtPRD1-B (389-812 aa), AtPRD1-C (813-962 aa), AtPRD1-D (963-1109 aa) and AtPRD1-E (1110-1330 aa). (b) The structural organization of the MTOPVIB and AtSPO11-2 proteins, indicating the two truncated fragments of MTOPVIB: 1-261 aa (Bergerat domain) and 228-493 aa (Transducer domain) and AtSPO11-2: 1-210 aa (CAP domain) and 170-383 aa (Toprim domain) used in the interaction assays.

Print to PDF without this message by purchasing novaPDF (http://www.novapdf.com/)

Figure S13.

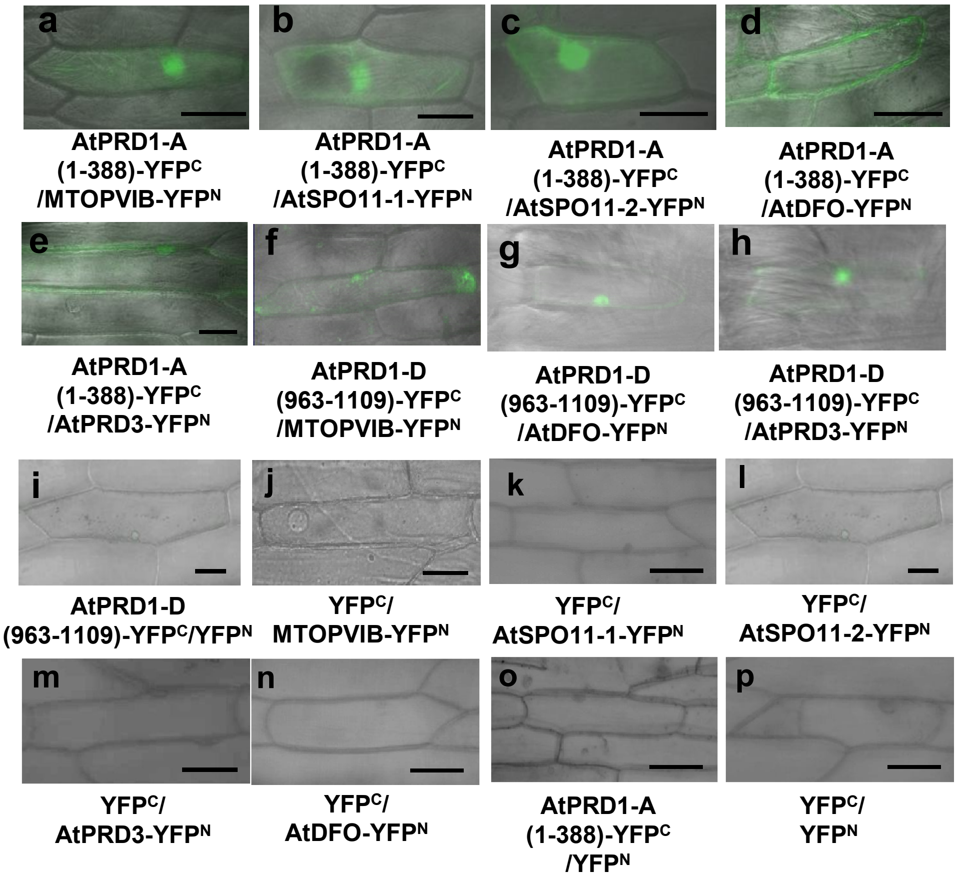

Figure S13. BiFC assays for interactions of AtPRD1-A and –D with Topo VI-like complex, AtPRD3, AtDFO in onion epidermal cells.

(a-i) showed that AtPRD1-A (1-388 aa) interacted with MTOPVIB (a), AtSPO11-1 (b), AtSPO11-2 (c), AtDFO (d) and AtPRD3 (e). (f-h) AtPRD1-D (963-1109 aa) interacted with MTOPVIB (f), AtDFO (g) and AtPRD3 (h). (i-p) The negative controls for (a-h). Bars= 50 μm.

Print to PDF without this message by purchasing novaPDF (http://www.novapdf.com/)
